# The metabolic analysis of psoriasis identifies the associated metabolites while providing computational models for the monitoring of the disease

**DOI:** 10.1007/s00403-017-1760-1

**Published:** 2017-07-10

**Authors:** Aigar Ottas, Dmytro Fishman, Tiia-Linda Okas, Külli Kingo, Ursel Soomets

**Affiliations:** 1 Department of Biochemistry, Institute of Biomedicine and Translational Medicine, University of Tartu, Ravila 14b, 50411 Tartu, Estonia; 2 Faculty of Science and Technology, Institute of Computer Science, University of Tartu, Tartu, Estonia; 3 Quretec OÜ, Tartu, Estonia; 4 University of Tartu, Tartu, Estonia; 5 Department of Dermatology, University of Tartu, Tartu, Estonia; 6 Clinic of Dermatology, Tartu University Hospital, Tartu, Estonia; 7 Centre of Excellence for Genomics and Translational Medicine, University of Tartu, Tartu, Estonia

**Keywords:** Metabolomics, Psoriasis, Targeted analysis, Untargeted analysis, Computational model

## Abstract

The majority of studies on psoriasis have focused on explaining the genetic background and its associations with the immune system’s response. The aim of this study was to identify the low-molecular weight compounds contributing to the metabolomic profile of psoriasis and to provide computational models that help with the classification and monitoring of the severity of the disease. We compared the results from targeted and untargeted analyses of patients’ serums with plaque psoriasis to controls. The main differences were found in the concentrations of acylcarnitines, phosphatidylcholines, amino acids, urea, phytol, and 1,11-undecanedicarboxylic acid. The data from the targeted analysis were used to build classification models for psoriasis. The results from this study provide an overview of the metabolomic serum profile of psoriasis along with promising statistical models for the monitoring of the disease.

**Electronic supplementary material:**

The online version of this article (doi:10.1007/s00403-017-1760-1) contains supplementary material, which is available to authorized users.

## Introduction

Psoriasis is an immune-mediated skin disorder, where in addition to visible scaly inflamed plaques on the skin, the joints and nails might also be affected. The cause of psoriasis remains uncertain, but it is known that a genetic predisposition accompanied with environmental factors, such as stress, smoking, and alcohol abuse, can lead to the development of the disease. Psoriasis patients are also at a higher risk for metabolic syndrome, cardiovascular diseases, and overall morbidity [14]. The past work on psoriasis has focused mainly on the genetical background and immunology of the disease with fewer papers published on metabolomics [6, 31]. Metabolomics is an emerging field in the omics family that concerns with the identification and quantitation of small molecules including amino acids, carbohydrates and their derivatives, biogenic amines, lipids, and more. Current work on the metabolomics of psoriasis is very limited with only a number of published papers. Kamleh and his colleagues have discovered changes in free-circulating amino acids, namely, arginine, proline, alanine, glutamate, aspartate, glycine, serine, and threonine, which levels are elevated in the plasma of patient psoriasis. The levels of amino acids revert to normal after the biological treatment with TNFα receptor blocker Etanercept [21]. In another study by Armstrong et al., higher concentrations of alpha ketoglutaric acid and glucuronic acid and lower levels of asparagine and glutamine were determined in psoriasis patients’ serum. In addition, patients with both psoriasis and psoriatic arthritis showed higher levels of lignoceric acid and a lower level of alpha ketoglutaric acid in the serum when compared to patients with psoriasis alone [4].

Metabolomics employs many different methods to analyze samples which vary depending on the medium, collection methods and time [25, 60]. Therefore, using varied approaches to measurements can yield in a higher coverage of available metabolites. In this study, serum samples from psoriasis patients and controls were analyzed using a targeted approach that analyses the concentrations of known metabolites, e.g., amino acids, lipids, biogenic amines, etc. and an untargeted profiling that helps to discover metabolites not included in the targeted method.

Metabolomics produces a lot of data for every measured sample which makes data interpretation challenging. Many different mathematical methods have been used including partial least squares discriminant analysis or principal component analysis (PCA) [15]. Using machine learning and algorithms for metabolomic profiling is a promising solution for data interpretation and automation [35, 41].

## Materials and methods

### Recruitment of volunteers

For untargeted analysis, a total of 40 volunteers were included in this study—20 diagnosed with plaque psoriasis and 20 age and sex-matched controls (13 men, 7 women, age range 20–75). For targeted analysis, the number of volunteers were expanded to 106—55 psoriasis patients (37 men, 18 women, age range 20–75) and 51 controls (15 women, 36 men, age range 23–75). The subjects with plaque psoriasis recruited for this study were patients in the Clinic of Dermatology at the University Hospital of Tartu. The participating subjects were diagnosed with plaque psoriasis by dermatologists. Psoriasis Area and Severity Index (PASI) scores were calculated ranging from 1 to 34 to allow the analysis of a wide disease span. Controls were recruited either from the same clinic or from the Clinic of Traumatology. Exclusion criteria for the patients and controls included any other skin diseases, diabetes, gout, hypertension, and the taking of prescription medication. Detailed questionnaires were filled out that covered age, sex, skin type, comorbidities, smoking status, and PASI scores.

### Blood sample collection and storing

Fasting blood samples were collected before breakfast in the morning into 5 ml Vacutainer (REF 367614) tubes that have micronized silica particles for the acceleration of the clotting process. The collected blood was left to clot for 1 h at room temperature after which it was centrifuged at 1300×*g* for 20 min. The supernatant serum was aliquoted into 300 µl fractions and placed in the freezer at −80 °C.

### Materials

HPLC-grade solvents [acetonitrile, water, and formic acid (FA)] were purchased from Sigma-Aldrich (Germany). For the targeted approach, an Agilent Zorbax Eclipse XDB C18, 3.0 × 100 mm, 3.5 µm with Pre-Column SecurityGuard, Phenomenex, C18, 4 × 3 mm was used with the Absolute*IDQ* p180 kit (Biocrates Life Sciences AG, Innsbruck, Austria). For chromatographic separation in the untargeted part, a SeQuant^®^ ZIC^®^-pHILIC (5 µm polymer) PEEK 150 × 4.6 mm metal-free HPLC column and ZIC^®^-pHILIC Guard column PEEK 20 × 2.1 m were used.

### Mass-spectrometric-targeted analysis

For targeted analysis, the serum samples were thawed on ice and prepared according to the specifications detailed in the Absolute*IDQ* p180 kit’s user manual. Shortly, 10 µl of serum was pipetted onto 96-well plate filter inserts, internal standards added, and samples dried under nitrogen and derivatized using phenylisothiocyanate. The samples were measured using a combination of flow injection analysis and through a C18 column. The prepared kit plate was analyzed on a QTRAP 4500 (ABSciex, USA) mass spectrometer which was coupled to an Agilent 1260 series HPLC (USA). The results from the analysis were quantified concentrations of different acylcarnitines, amino acids, biogenic amines, hexose, glycerophospholipids, sphingolipids, and ratios of different metabolites.

### Mass-spectrometric-untargeted analysis

Blood samples were left to thaw on ice. 100 µl of serum was pipetted into a new Eppendorf tube, where 400 µl of acetonitrile was added for protein precipitation. The tube was vortexed vigorously for 2 min and left for 15 min at room temperature. The mixture was centrifuged for 15 min at 15,800×*g* and 4 °C. The supernatant was transferred to a clean tube, the samples were randomized, and 10 µl of sample was used for analysis. A Shimadzu HPLC (Japan) was coupled to a 3200 QTRAP (ABSciex, USA) mass spectrometer, where the parameters were as follows: solvents used were acetonitrile + 0.1% FA, water + 0.1% FA, runtime 62 min, gradient flow rate 0.3 ml/min from 80% acetonitrile to 20% in 32 min, to 5% in 1 min, at 5% for 8 min, then to 100% in 5 min, at 100% for 8 min, then re-equilibration at 80% for 8 min. The turbo spray’s curtain gas was set to 10 au, collision gas to “High”, ionspray voltage to 4500 V, temperature to 300 °C, declustering potential to 20 V, entrance potential to 10 V, and collision energy to 10 V in measurements and 20, 30, or 40 V in fragmentation analysis. The samples were measured in both positive and negative mode from 50 to 1500 mass-to-charge ratios (*m*/*z*). Fragmentation analysis was performed using the same settings and column but in Enhanced Product Ion mode for the statistically significant masses.

### Identification of metabolites

The spectra from the fragmentation analysis were compared to spectra from public databases METLIN [52], HMDB [59], MassBank [18], and LipidMaps [11]. A compound was considered identified when the fragmentation spectra, its peaks, and relative heights of peaks of a certain m/z were identical to a spectrum from an online database.

### Data processing

For the untargeted analysis, the acquired .wiff files were converted to .mzXML using the MSConvert software [10]. The data were analyzed in RStudio version 0.98.501 [55], where peaks were extracted using XCMS [53] and further processed using mzMatch.R [48] which included the combining of biological replicates, Reproducibility Standard Deviation filtering, retention time correction, blank filtering, gap filling, filtering on the number of detections (minimum of six), and matching of related peaks. After data processing, a Wilcoxon–Mann–Whitney test was performed to determine the peaks and their corresponding retention times that differ statistically differently between subjects with plaque psoriasis and controls. The differentiating *m*/*z*-s were selected for fragmentation analysis. For targeted analysis, a Wilcoxon–Mann–Whitney test was used to determine which metabolites differ statistically significantly between controls and subjects with plaque psoriasis.

Principal component analysis was applied for the visualization of general differences between the groups on data from both targeted and untargeted analysis measurements [20].

### Metadata and pre-processing

The collected data are suitable for the purposes of modeling, since the disease and control classes are of equal size. Although there are twice as many males, the distribution of ages and diagnosis is very similar between both sexes (Figure S1). Prior to analysis, metabolites, which showed zero or close to zero variance between samples, were removed. Scaling and centering was applied to all values in the data set.

### Modeling

For the accurate discrimination between psoriasis patients and controls, we used a popular machine learning and bioinformatics field’s method—random forest algorithm [17]. It has been shown to work well in a wide variety of biological problems including the identifying of regulatory regions [39], classifying metabolomics data [1], and selecting highly reliable biomarkers for the diagnosis of Alzheimer’s disease [36]. Random forest is an extension of another machine learning technique—decision tree [38] which builds a series of conditions (decisions) that best describe the underlying distribution of classes. Generated by the algorithm the series of decisions can be visualized as a tree-like structure. Random forest is an ensemble of decision trees built using random subsets of original training data.

### Feature selection and cross validation

To select only relevant metabolite features for our classifications, we used three feature selection methods. Two were wrapper methods: a genetic algorithm [33] and a recursive feature elimination and one was a filter method [46]. All three methods were used in parallel to cross-check the results. All of them used external cross validation [3] to avoid unwanted bias that could be introduced by aggressive feature selection procedures.

Due to the lack of training data, the use of fivefold cross-validation strategy was applied as suggested by Ambroise et al. [3]. We only report average area under receiver operating characteristic (AUROC) values for our feature selection models. We used random forest with all three feature selection techniques. Modeling was implemented using R version 3.3.1 and package ‘caret’ 6.0.71 [27].

## Results

Metabolites from the targeted approach that differ statistically significantly in the serum of subjects with plaque psoriasis from controls are shown in Table 1. We found the differences in acylcarnitine levels, mainly in the concentrations of nonaylcarnitine (C9), dodecanoylcarnitine (C12), decadienylcarnitine (C10.2), and pimelylcarnitine (C7.DC) which are all lower in concentrations in psoriasis subjects’ serum.

**Table 1:** Statistically significantly different metabolites and their ratios from targeted analysis

Metabolite abbreviation	Metabolite	*p* value	Psoriasis mean μM ± SD	Control mean μM ± SD
Met.SO	Methioninesulfoxide	6.06E−06	0.88 ± 0.37	0.51 ± 0.27
Met.SO…Met	Fraction of sulfoxidized methionine of the unmodified methionine pool	2.65E−05	0.04 ± 0.02	0.02 ± 0.01
C9	Nonaylcarnitine	0.002	0.04 ± 0.01	0.05 ± 0.01
Glu	Glutamate	0.002	92.85 ± 66.43	49.06 ± 22.76
Cit…Orn	Ratio of citrulline to ornithine	0.002	0.37 ± 0.13	0.44 ± 0.12
C2…C0	ratio of acetylcarnitine to free carnitine	0.004	0.17 ± 0.08	0.22 ± 0.08
X.C2.C3…C0	Ratio of short-chain acylcarnitines to free carnitine	0.005	0.18 ± 0.08	0.23 ± 0.08
PC.aa.C36.6	Phosphatidylcholine diacyl C36:6	0.006	0.68 ± 0.27	0.89 ± 0.33
Total.AC…C0	Ratio of esterified to free carnitine	0.006	0.25 ± 0.1	0.31 ± 0.1
PC.ae.C38.0	Phosphatidylcholine acyl-alkyl C38:0	0.007	1.73 ± 0.5	2.17 ± 0.69
C7.DC	Pimelylcarnitine	0.011	0.019 ± 0.006	0.024 ± 0.008
Orn	Ornithine	0.011	99.79 ± 29.44	82.28 ± 20.85
PC.ae.C40.6	Phosphatidylcholine acyl-alkyl C40:6	0.011	3.39 ± 0.99	4.02 ± 1.01
Putrescine…Orn	Ratio of putrescine to ornithine	0.013	0.001 ± 0.001	0.002 ± 0.001
PC.aa.C36.5	Phosphatidylcholine diacyl C36:5	0.019	24.78 ± 13.25	34.34 ± 19.98
Phe	Phenylalanine	0.026	82.91 ± 18.96	72.46 ± 13.51
[C16 + C18]/C0	Ratio of long-chain acylcarnitines to free carnitine	0.027	0.004 ± 0.001	0.005 ± 0.001
C12	Dodecanoylcarnitine	0.036	0.1 ± 0.036	0.124 ± 0.051
C10.2	Decadienylcarnitine	0.044	0.069 ± 0.021	0.076 ± 0.02

Phosphatidylcholine diacyls (PC aa) C36:5/C36:6 and phosphatidylcholine acyl-alkyls (PC ae) C38:0/C40.6 all showed higher levels in controls’ serum. Statistically significantly differing levels of amino acids were found to be for glutamate (Glu), ornithine (Orn), phenylalanine (Phe), and methioninesulfoxide (Met.SO). Amino acid concentrations in samples were statistically significantly higher in psoriasis subjects compared to controls. Ratios of acylcarnitine to free carnitine (C2…C0), short-chain acylcarnitines to free carnitine (X.C2.C3…C0), citrulline to ornithine (Cit…Orn), esterified to free carnitine (Total.AC…C0), putrescine to ornithine (Putrescine…Orn), and long-chain acylcarnitines to free carnitine ([C16 + C18]/C0) were all statistically significantly higher in controls, whereas the levels of the fraction of sulfoxidized methionine of the unmodified methionine pool (Met.SO…Met) were higher in psoriasis patients.

All metabolites which levels differed statistically significantly in the untargeted measurements are shown in Table 2. Identification was successful for 12 metabolites out of 22 (supplementary Figures S2–S13). All of the discovered metabolites showed higher levels in samples of psoriasis patients. They include urea, taurine, phytol, 1,11-undecanedicarboxylic acid, glycerophosphocholines PC(16:0/18:2), PC(18:1/0:0), PC(16:0/18:1), PC(16:0/0:0), PC(20:4/0:0), PC(18:1/0:0), and phosphatidylethanolamine PE(20:4/0:0).

**Table 2:** Statistically significantly different *m*/*z*-s from untargeted analysis

	Mass-to-charge ratio	*p* value	Intensity levels higher in psoriasis or controls?	Metabolite
Negative ionization	189	2.80E−07	Psoriasis	No match in databases
	802.5	8.28E−05	Psoriasis	PC(16:0/18:2) + FA
	556.32	2.91E−04	Psoriasis	PC(18:1/0:0)
	129.17	4.82E−05	Psoriasis	No match in databases
	249	1.46E−04	Psoriasis	No match in databases
	198	8.36E−05	Psoriasis	No match in databases
	325.5	1.03E−04	Psoriasis	No match in databases
	249	1.30E−04	Psoriasis	No match in databases
	243.12	9.73E−08	Psoriasis	1,11-Undecanedicarboxylic acid
Positive ionization	760.56	7.91E−07	Psoriasis	PC(16:0/18:1)
	496.38	8.15E−07	Psoriasis	PC(16:0/0:0)
	159	3.07E−06	Psoriasis	No match in databases
	126	1.59E−03	Psoriasis	Taurine
	544.38	1.31E−06	Psoriasis	PC(20:4/0:0)
	282	1.58E−06	Psoriasis	No match in databases
	297	1.10E−05	Psoriasis	No match in databases
	297.059	4.30E−03	Psoriasis	Phytol
	522.36	2.79E−03	Psoriasis	PC(18:1/0:0)
	120	3.94E−06	Psoriasis	No match in databases
	502.38	0.0108	Psoriasis	PE(20:4/0:0)
	679.5	5.54E−08	Psoriasis	No match in databases
	60.69	1.23E−05	Psoriasis	Urea

**Fig. 1:**
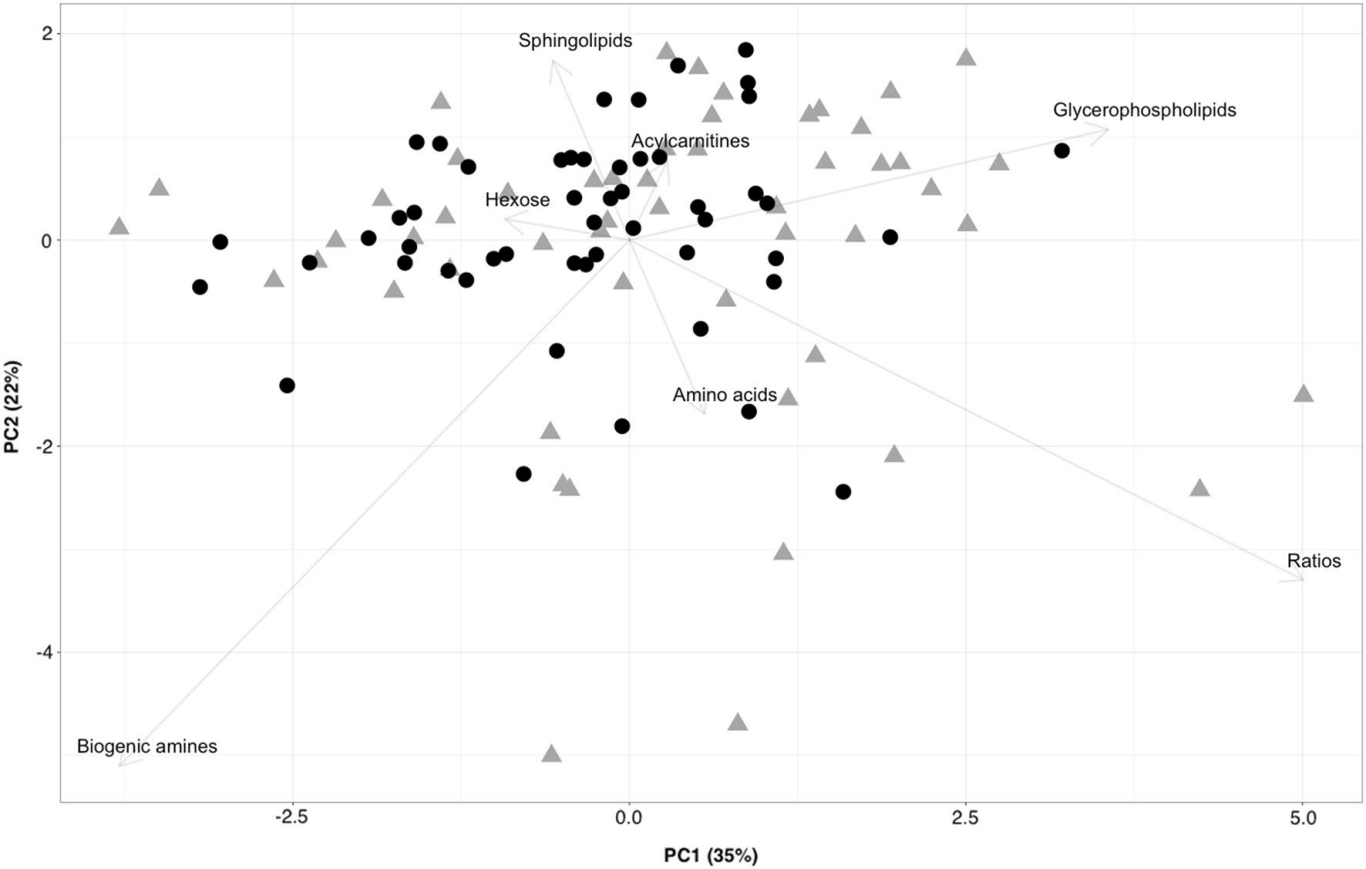
PCA plot of the targeted analysis. Psoriasis samples are marked as *gray triangles* and control samples as *black circles*. The metabolite groups responsible for the separation are marked at the end of the *arrows*. *X* and *Y* axes represent the percentage of variability explained by principal components one and two

**Fig. 2:**
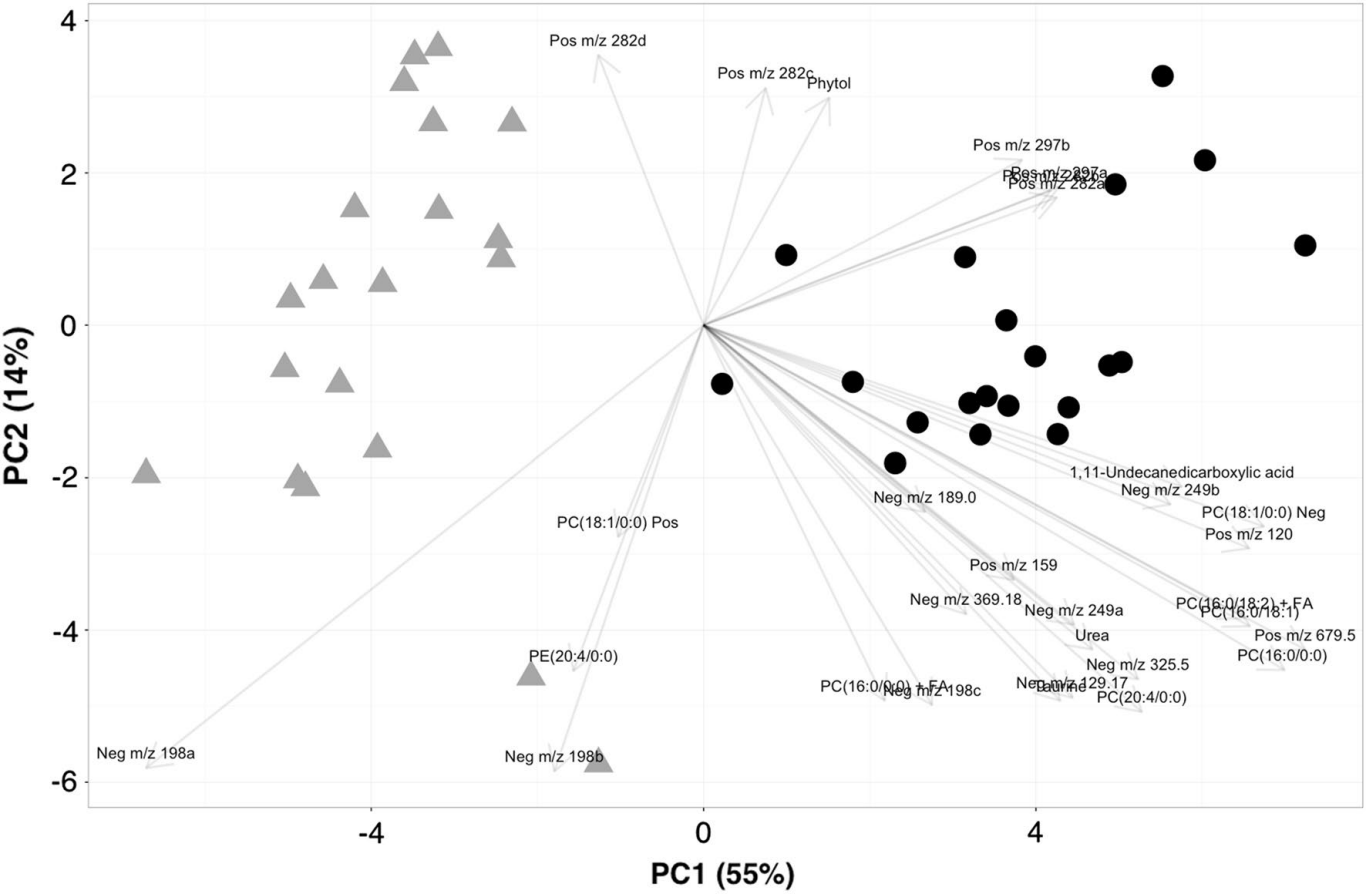
PCA plot of the untargeted analysis. Controls are shown as *gray triangles,* while psoriasis patients are marked as *black circles*. The metabolites responsible for the separation are shown at the end of the *arrows*. *X* and *Y* axes represent the percentage of variability explained by principal components one and two

PCA plot from targeted metabolites which are summed based on their metabolite classes (Fig. 1) shows two overlapping clusters of controls and psoriasis groups. The group clustering is best observed along principal component one that accounts for 35% of variability. For principal component one, the metabolites responsible for the clustering of groups are biogenic amines, glycerophospholipids, and metabolite ratios. The combined PCA plot of the positive and negative ionization analyses from the untargeted approach displays very clear clustering of the groups on the axis of PC 1 that accounts for 55% of variability (Fig. 2). As expected, there is not a single metabolite that is solely responsible for the clustering of groups in the PCA plot, but a combination of many metabolites both identified and unidentified. The better clustering can be explained by the inclusion of fewer metabolites due to the lower accuracy of the mass spectrometer in addition to the application of many filtering techniques used in data pre-processing.

A random forest classifier with three different feature selection methods was trained. Each combination of random forest and feature selection method has been trained on the whole data set using fivefold cross validation to ensure fair model performance estimate.

**Fig. 3:**
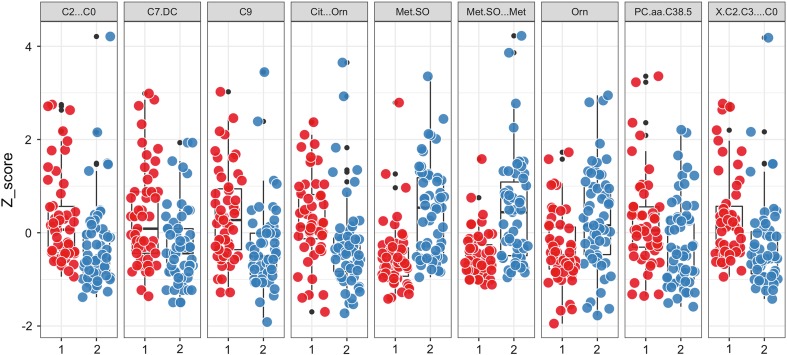
Distribution of standardized signals for nine metabolites overlapping in all three modeling methods. *Red dots* represent standardized concentrations for psoriasis patients, while *blue ones* represent controls

We report that with recursive feature elimination, our random forest model achieved 0.86 AUC, 0.77 sensitivity and 0.74 specificity averaged across five repetitions. With this method, 15 metabolites were selected into a final model. Feature selection using internal importance measures that applied inside random forest model yielded 46 metabolites and 0.85 AUC resampling performance with sensitivity and specificity 0.77 and 0.74 correspondingly. Finally, the best model that used genetic algorithm for feature selection kept 90 features and achieved 0.85 AUC. 9 metabolites/ratios was selected by all three methods and these are Met.SO, Cit…Orn, Met.SO…Met, X.C2.C3…C0, C2…C0, C9, Orn, C7.DC, and PC.aa.C38.5. Figure 3 shows the difference in concentrations of nine overlapping metabolites between healthy subjects and psoriasis patients. All of these differences are statistically significant with the exception of PC.aa.C38.5, whereas the maximum Wilcoxon test *p* value equals 0.011 for Orn and the minimal 6.06E−06 for Met.SO.

## Discussion

The elevated levels of amino acids in the serum of psoriasis patients established in this present study can be linked to the higher demand for amino acids in the hyperproliferative epidermis, where de novo synthesis of proteins is upregulated and the rate of mitosis in basal keratinocytes is increased compared to non-lesional skin [37]. In our study, all of the statistically significantly different levels of amino acids are from the non-essential amino acid pool with the exception of phenylalanine. Ornithine is part of the urea cycle and its levels in the serum were discovered to be over-expressed in psoriatic patients. Arginase I (EC 3.5.3.1) catalyzes arginine hydrolysis to urea and ornithine and has been demonstrated to be a limiting factor for cell proliferation [58]. Higher activity of arginase has been demonstrated in psoriatic skin [2] which can be linked to the hyperproliferating keratinocytes and higher concentrations of ornithine. The end product of the ornithine cycle is urea that we discovered in the untargeted analysis to have higher levels in psoriatic patients’ serum. The ratio of the fraction of symmetrically dimethylated arginine of the unmodified arginine pool nor ADMA showed statistically significant differences in serums (*p* values 0.35 and 0.31, respectively), thus contradicting the recent results by Bilgic et al. [5]. This illustrates the variability of psoriasis cohorts and the necessity for an even larger cohort studies to confirm the results of either study. Glutamate which level is significantly higher in psoriasis patients samples (*p* = 0.002) can be converted by glutamine synthetase (EC 6.3.1.2) to glutamine which is subsequently converted by carbamoyl-phosphate synthetase (EC 6.3.4.16) to carbamoyl-P which is then converted by ornithine carbamoyltransferase (EC 2.1.3.3) to citrulline that is an essential component of the urea cycle [23]. It can be hypothesized that in addition to the overproduction of the urea cycle intermediates in psoriatic patients, the increase in concentrations of said metabolites come from changes in proteins that are deiminated (or citrullinated) in the skin mainly K1 [49], K10, and filaggrin proteins [22]. The decreased deimination of psoriatic cornified cell layer proteins has been shown for K1 [19]. Fewer cells that express K10 were detected in lesional skin [34]. Filaggrin, filaggrin-2, and their mRNAs in psoriatic skin samples were found to be significantly reduced [32]. Since less citrulline is produced in psoriatic skin, a cumulation of urea cycle intermediates can be explained. The ratio of citrulline to ornithine is lower in psoriasis patients, thus indicating the lower activity of ornithine carbamoyltransferase and the cumulation of ornithine. In addition, the ratio of putrescine to ornithine is lower in psoriasis patients showing a reduction in the activity of ornithine decarboxylase (EC 4.1.1.17) and to overall changes in the urea cycle. These findings correlate well with Kang et al. [24] who also noted the upregulation of metabolites in the urea cycle. Phenylalanine is a non-essential amino acid meaning that its uptake is dependent on diet. Phenylalanine is hydroxylated to tyrosine by phenylalanine hydroxylase (PAH, EC 1.14.16.1). PAH activity has been shown to be lower in psoriasis patients [26] and increases after UVB-light exposure [47], thus lowering the amount of Phe in the serum. Although the role of excess Phe in psoriasis is unclear, a connection with UV therapy and its effect can be noted.

Methioninesulfoxide (Met.SO) is the oxidized form of methionine that reacts with free radicals and goes through the oxidation process [7]. The higher concentration of Met.SO and the ratio for the fraction of sulfoxidized methionine of the unmodified methionine pool are both indicative of oxidative stress in psoriasis patients’ serum.

Carnitine is synthesized from lysine and methionine [23] and has a variety of functions that include the transport of different fatty acids to mitochondria for branched α-keto acid oxidation, mitochondrial fatty acid oxidation, and trapping of acyl-CoA metabolites that may impair gluconeogenesis, the citric cycle, and the urea cycle [40] among many other functions. In omnivores, up to 75% percent of carnitine comes from dietary sources or can be from endogenous origins in the case of strict vegetarians [42]. In healthy individuals, up to 80% of carnitine from food is absorbed [43]. The lower concentration values of various carnitines and acylcarnitines found in psoriasis patients can either be explained by the unlikely change in diet that is less abundant in dairy products, fish, meat, and poultry or the increase in fatty acid oxidation in lesional skin due to the increased energy consumption of rapidly proliferating cells. Caspary et al. have shown the latter [9], where the increased activity of carnitine palmitoyltransferase-1 (CPT-1) was demonstrated in lesional skin. CPT-1 is the enzyme responsible for the rate of transport of long-chain fatty acids into mitochondria. This correlates well with our results that show a significant decrease in the concentrations of C9, C12, C10.2, and C7.DC in psoriasis subjects compared to controls. On the opposite, CPT-1 and CPT-2 deficiency has been shown to increase the concentrations of C14.1, C12, C16, C18:1, and C0 [50]. In addition to the changes in circulating carnitine levels, the ratios C2…C0, X.C2.C3…C0 and [C16 + C18]/C0 are all indicators of overall β-oxidation activity. Since the ratios are all higher in controls, it might be a sign of altered lipid use activity in the energy production in psoriasis.

The higher rate of proliferation for skin cells could also explain the lower concentrations of phosphatidylcholines in psoriasis patients’ blood serum that are essential parts of cell membranes. In targeted analysis, all of the phosphatidylcholines had lower concentrations in subjects with psoriasis. PC-s from untargeted analysis, however, had higher values in psoriasis patients’ serums which can be explained by the different methods and machinery used in the different approaches. The untargeted method strongly favors the solubility of lipophilic molecules, thus making some metabolites statistically different that were not in the targeted approach. LysoPC-s have been shown to induce the chemotaxis of T-lymphocytes [45], thus fueling the constant inflammation in the epidermis. A higher concentration of choline which is an essential precursor for phosphocholines has been noted in lesional psoriatic skin [51].

In targeted analysis, taurine had a higher concentration in psoriasis patients (122.6 µM) compared to controls (107.4 µM), but the change was not significant (*p* = 0.24). In untargeted analysis, taurine was also higher in psoriasis subjects and the change was considered statistically significant (*p* < 0.00159). Taurine is considered as a semi-essential amino acid that is not incorporated into proteins, but has different biological roles including the regulation of cell volume, anti-oxidative, anti-apoptotic, and anti-inflammatory effects [28]. Higher concentrations of taurine were measured in psoriatic skin compared to uninvolved skin [51].

Phytol is the carbon side chain of chlorophylls that is released by bacteria present in the gut of ruminants’ intestines. From there, it becomes widely available in the adipose tissue and dairy products of the animal [12]. This branched-fatty alcohol has been shown to activate peroxisome proliferator-activated receptor α in the liver followed by an effect on the control of lipid abnormalities in diabetes, obesity, and hyperlipidemia [13]. In addition, phytol is converted into phytanic acid in the liver [57]. Phytanic acid was demonstrated to increase oxidative stress and reduce the antioxidant potential in rats [29]. Alpha-methylacyl-CoA racemase (AMACR) is responsible for the regulation and metabolism of dietary branched-chain lipids [30]. In summary, it can be hypothesized that in psoriasis, the patients might have alpha and beta oxidation deficiencies which helps to explain the abnormal levels of acylcarnitines in the serum. The increase in the levels of phytol and phytanic acid contributes to oxidative stress and the continued inflammation of the skin in psoriatic patients. The single nucleotide polymorphism of AMACR in patients with psoriasis could also contribute to the increased level of phytol but that remains to be shown. Currently, a reduction of red meat and dairy products could potentially lower phytol levels in the serum. A meta-analysis has shown that dietary regulation and weight loss are important in achieving PASI 75 scores [56]. A reduced intake of phytol from dairy products and red meat might certainly have an effect on that.

An excess of 1,11-undecanedicarboxylic acid has been found in patients with Zellweger syndrome and adrenoleukodystrophy [44]. 1,11-undecanedicarboxylic acid together with phytanic acid could be indicative of peroxisome disorders, but the exact role remains unclear. Peroxisome proliferator-activated receptor-γ (PPARγ) has been demonstrated to be responsible for the regulation of lipid metabolism and glucose homeostasis along with cell differentiation and growth regulation [54]. Since the activity of PPARγ is lower in psoriatic skin [16], then higher concentrations of phytol, 1,11-undecanedicarboxylic acid, and different phosphatidylcholines could be a direct result of that.

Applying predictive models is still very new, but could 1 day be used routinely in the clinic for a better monitoring of the treatment or to provide help with diagnosis. The models we reported could very well be used for both. Building classification models are simpler, since it is a binary problem, but for the PASI regression model, the problem of measuring PASI scores by different physicians arises. It has been reported that an intra-class correlation coefficient of 0.804 was achieved when different doctors measured PASI scores on the same patients [8], so a more precise method of measurement, e.g., on the molecular level, could provide better treatment monitoring options.

In conclusion, our analysis demonstrated that patients with psoriasis have an impaired amino acid and lipid metabolism and a disbalance in the components of the bilipid cellular membrane. These findings will help to understand the pathogenesis of the disease which could lead to the better treatment of patients in the clinic. Our reported models and the discovered metabolites could be useful in helping to diagnose plaque psoriasis or monitor treatment.

## Electronic supplementary material

Below is the link to the electronic supplementary material.

Supplementary material 1 (PDF 1122 kb)
