# Supplementary material for: The metabolic analysis of psoriasis identifies the associated metabolites while providing computational models for the monitoring of the disease
Source: Arch Dermatol Res. 2017 Jul 10;309(7):519–28. doi: 10.1007/s00403-017-1760-1 (PMC5577063; doi:10.1007/s00403-017-1760-1)
Supplement: Supplementary file 1 — Supplementary material 1 (PDF 1122 kb) [file 403_2017_1760_MOESM1_ESM.pdf]

THE METABOLIC ANALYSIS OF PSORIASIS IDENTIFIES THE ASSOCIATED METABOLITES WHILE PROVIDING COMPUTATIONAL MODELS FOR THE MONITORING OF THE DISEASE

Archives of Dermatological Research

Aigar Ottas, Dmytro Fishman, Tiia-Linda Okas, Külli Kingo, Ursel Soomets

Corresponding author: Aigar Ottas

University of Tartu, Estonia.

e-mail: aigar.ottas@ut.ee

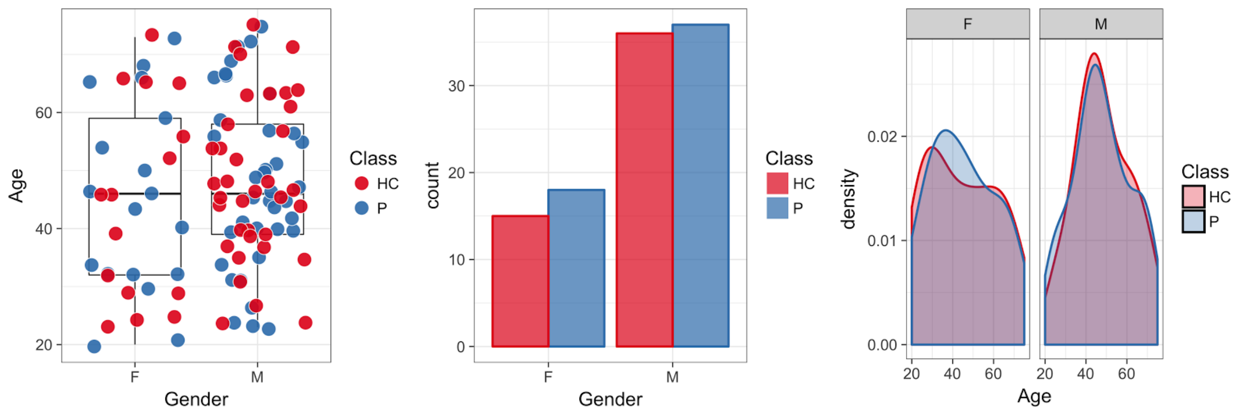

Figure S1. The distribution of gender, age and class variables. Psoriasis samples are marked with blue color and controls with red. Although males are overrepresented, the distribution of class and age are comparable between sexes.

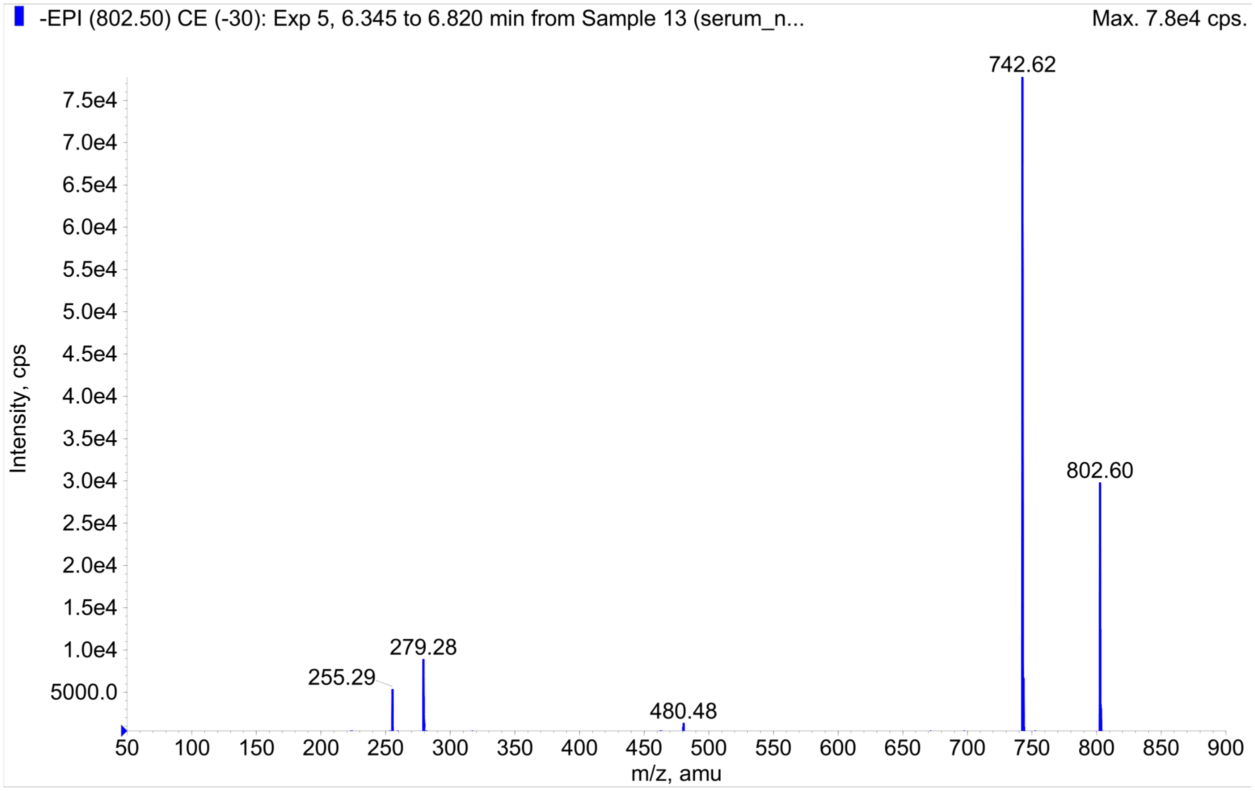

Figure S2a. Fragmentation spectrum of m/z 802.5 in negative ionization.

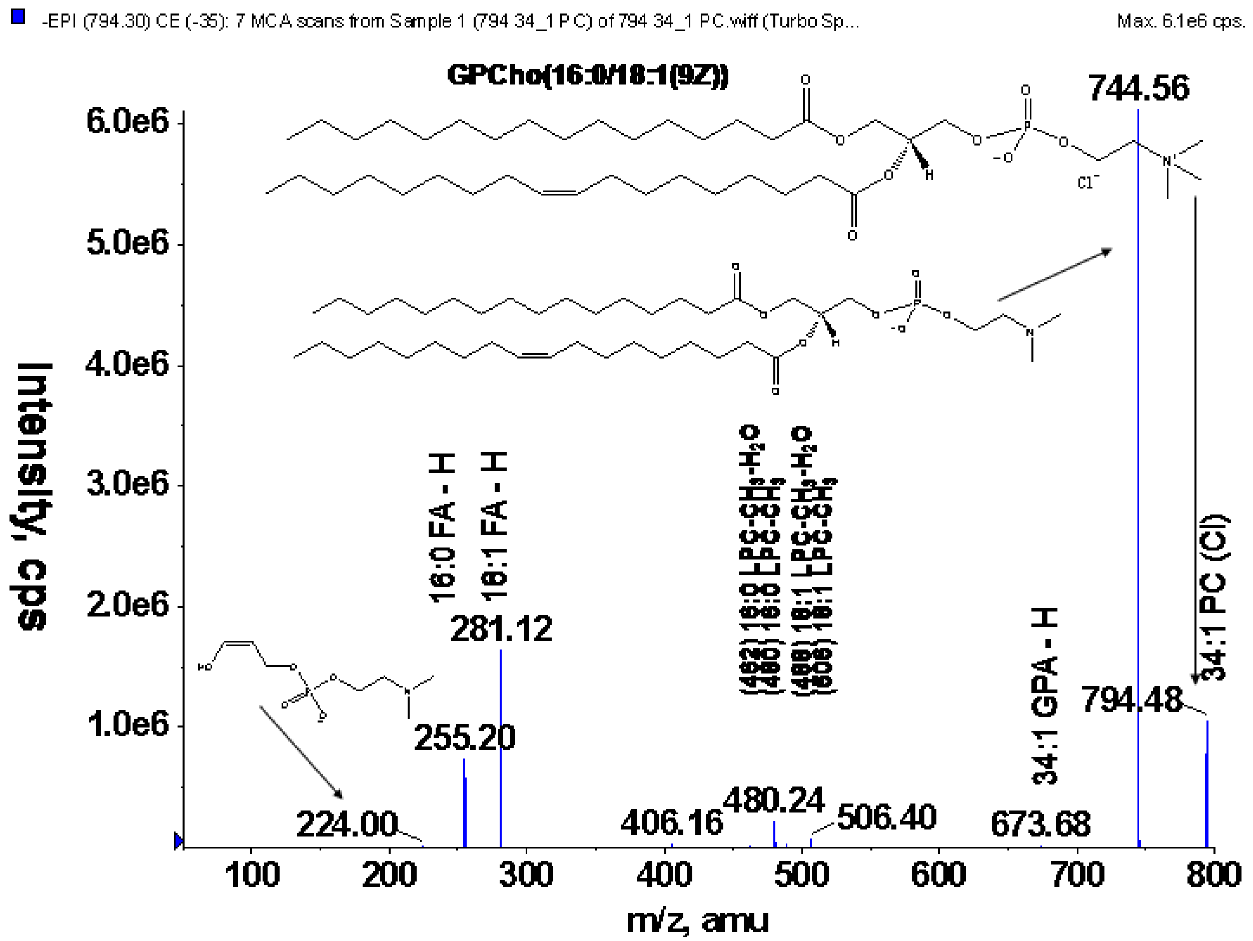

Figure S2b. Fragmentation spectrum of PC(16:0/18:1) in negative ionization.

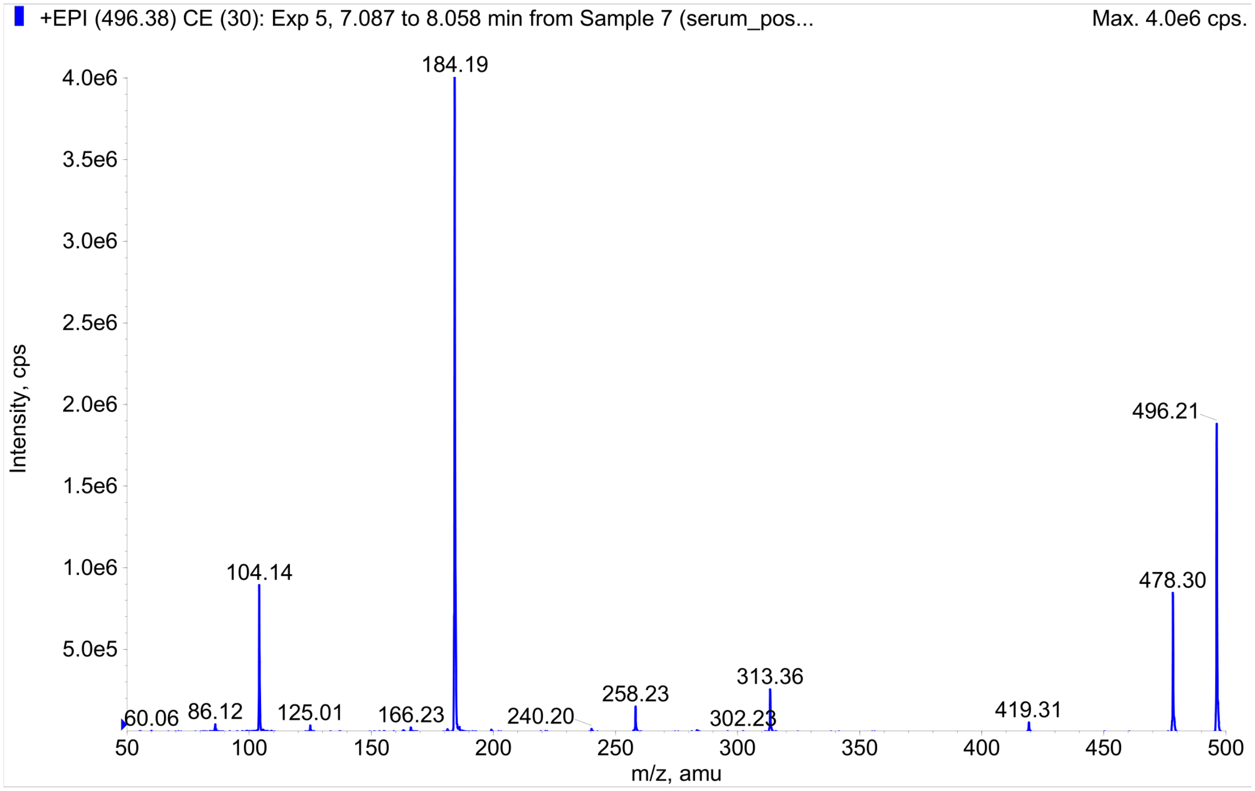

Figure S3a. Fragmentation spectrum of m/z 496.38 in positive ionization.

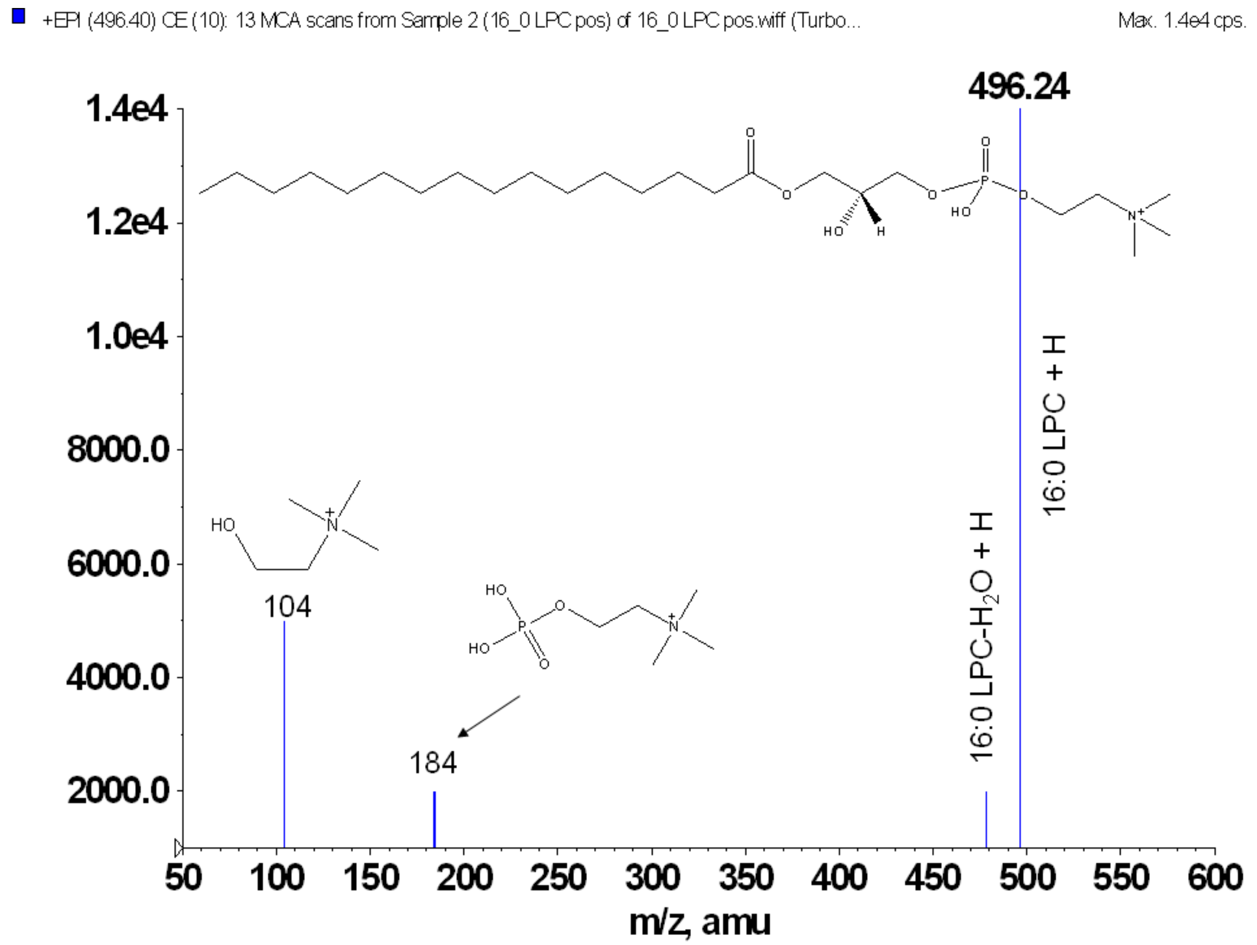

Figure S3b. Fragmentation spectrum of PC(16:0/0:0) in positive ionization

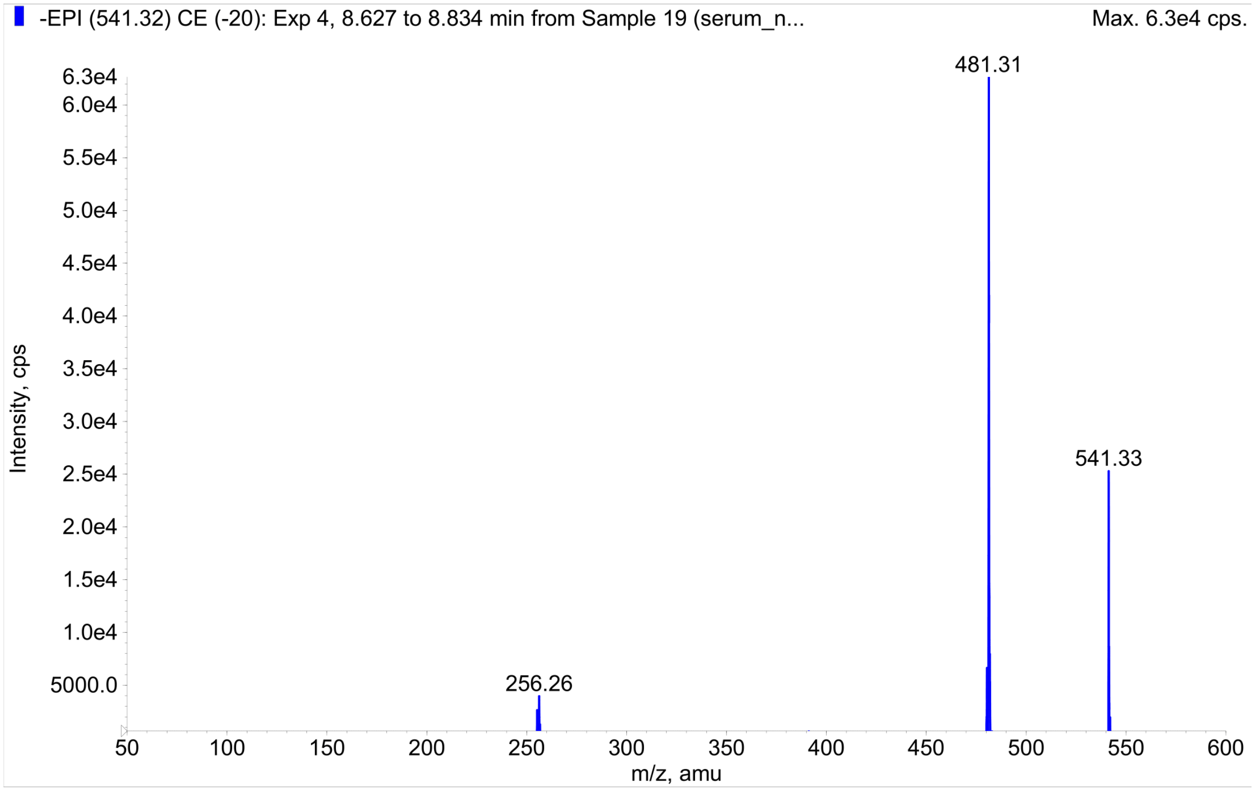

Figure S4a. Fragmentation spectrum of m/z 541.32 in negative ionization.

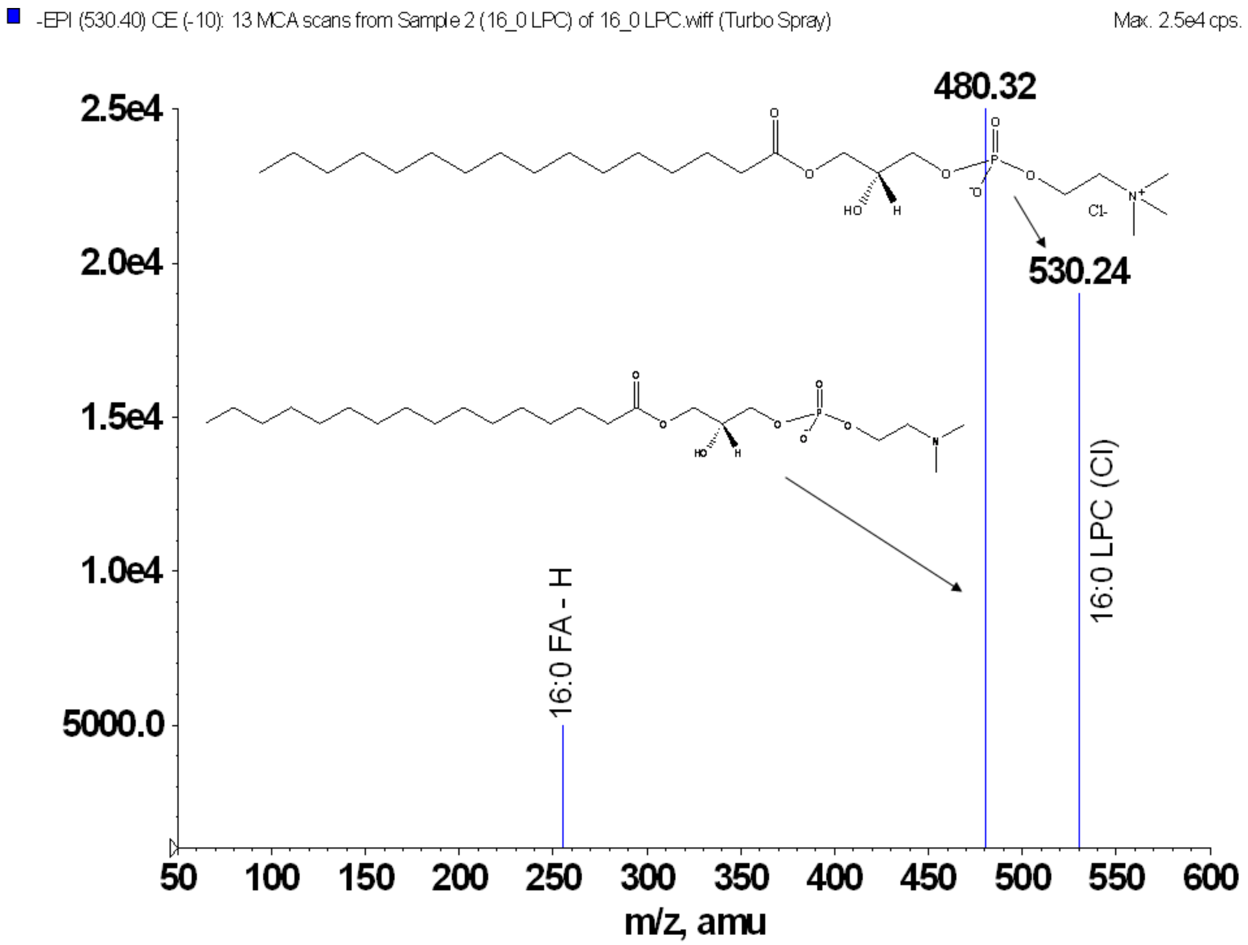

Figure S4b. Fragmentation spectrum of PC(16:0/0:0) in negative ionization.

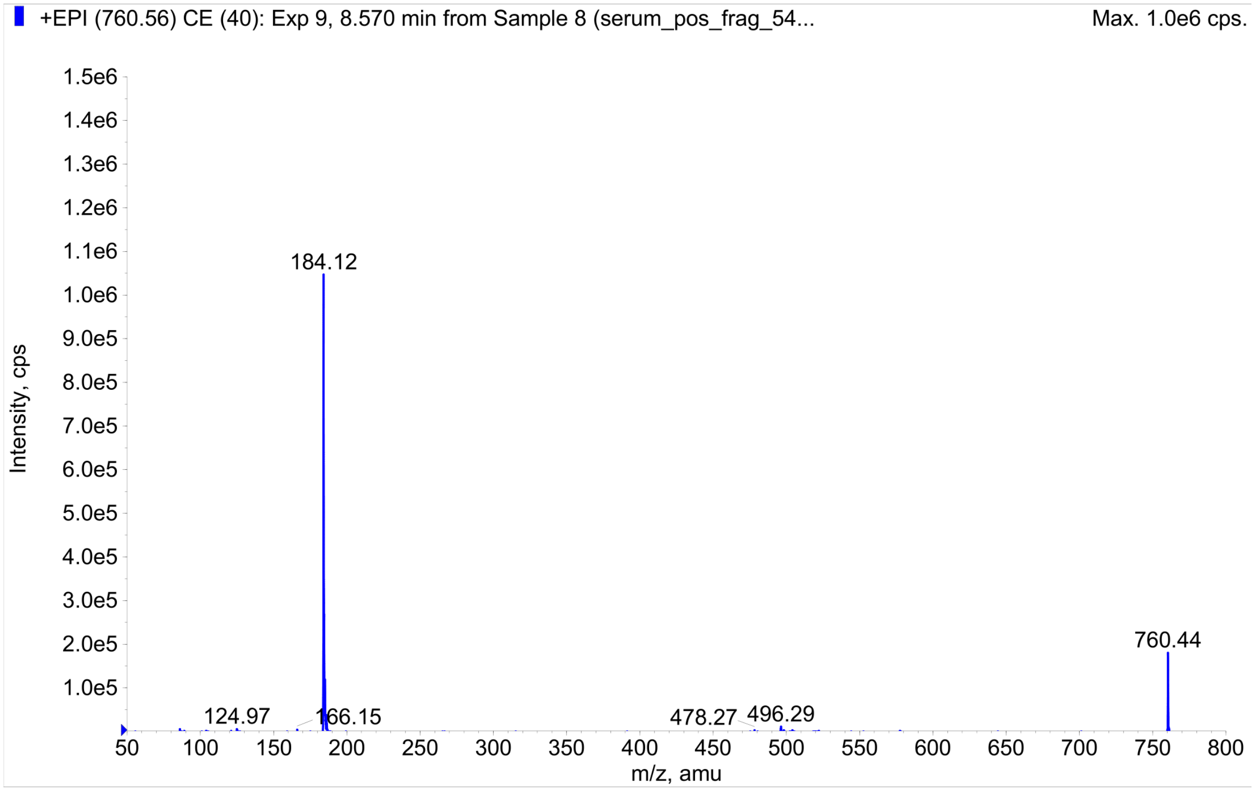

Figure S5a. Fragmentation spectrum of m/z 760.56 in positive ionization.

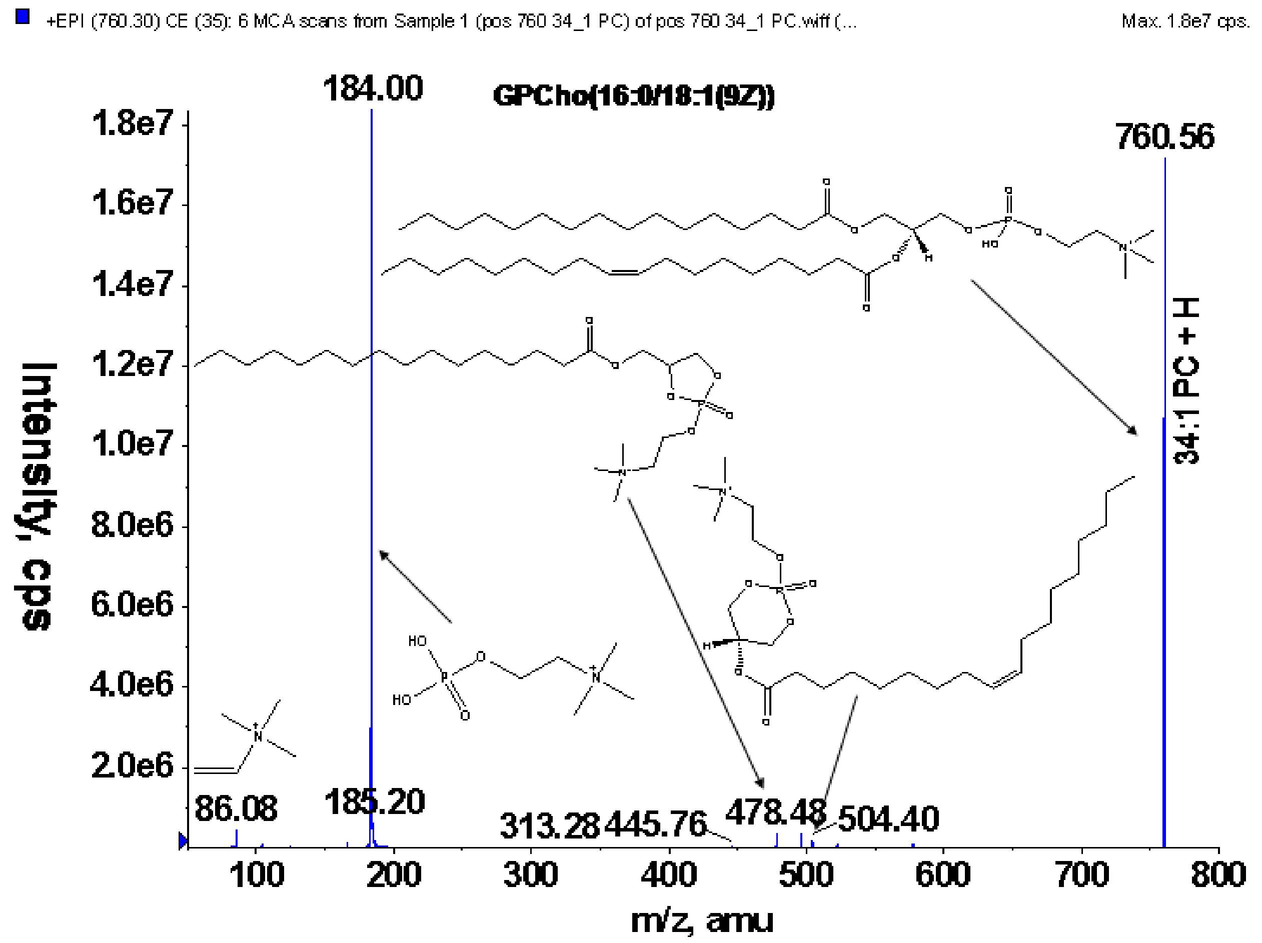

Figure S5b. Fragmentation spectrum of PC(16:0/18:1) in positive ionization.

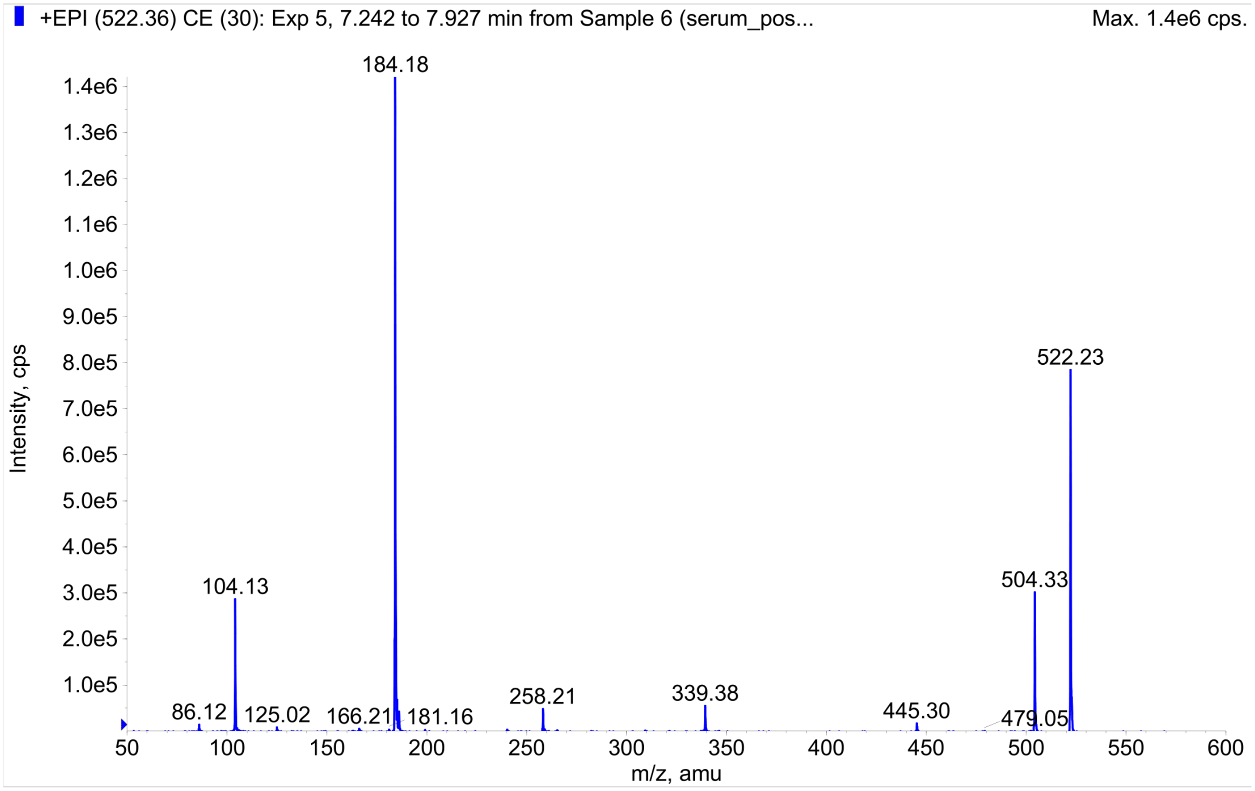

Figure S6a. Fragmentation spectrum of 522.36 m/z in positive ionization.

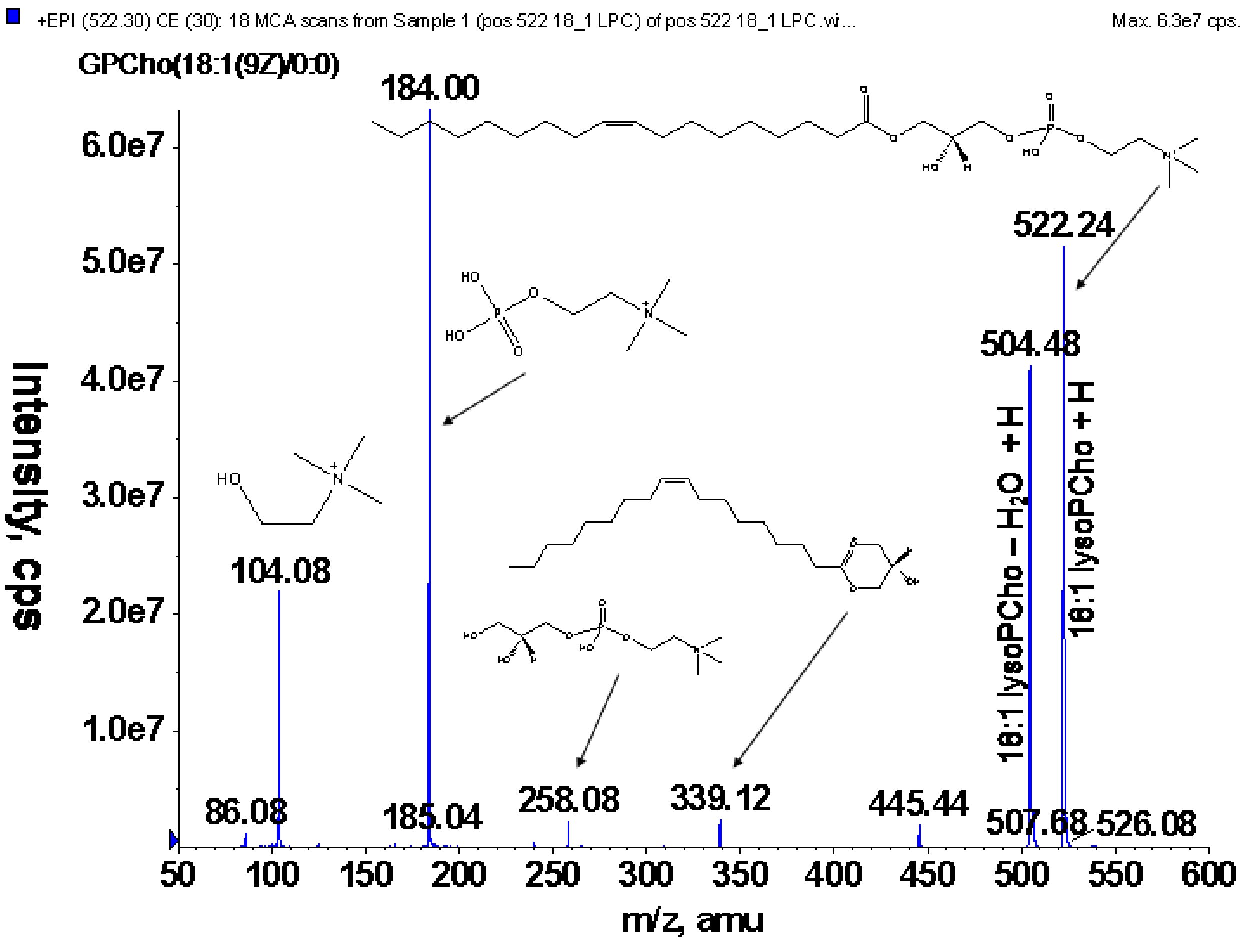

Figure S6b. Fragmentation spectrum of PC(18:1/0:0) in positive ionization.

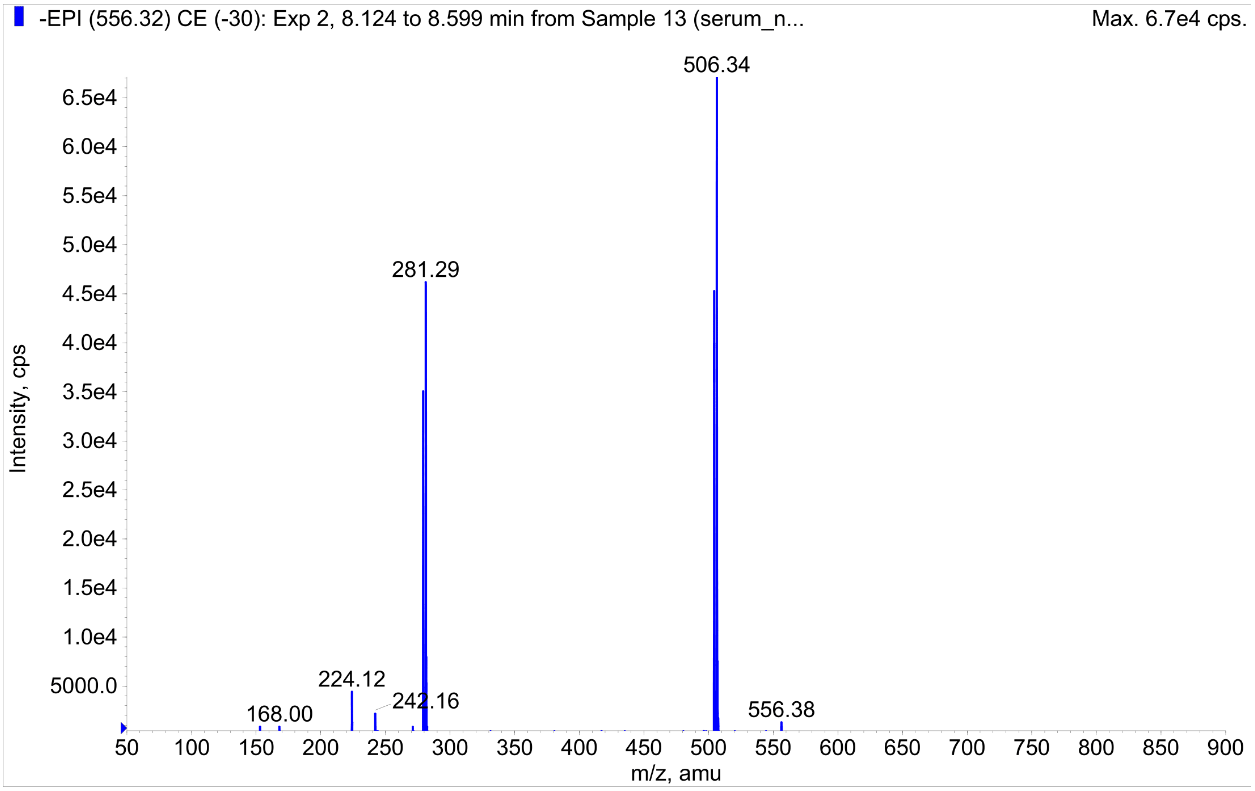

Figure S7a. Fragmentation spectrum of m/z 556.32 in negative ionization.

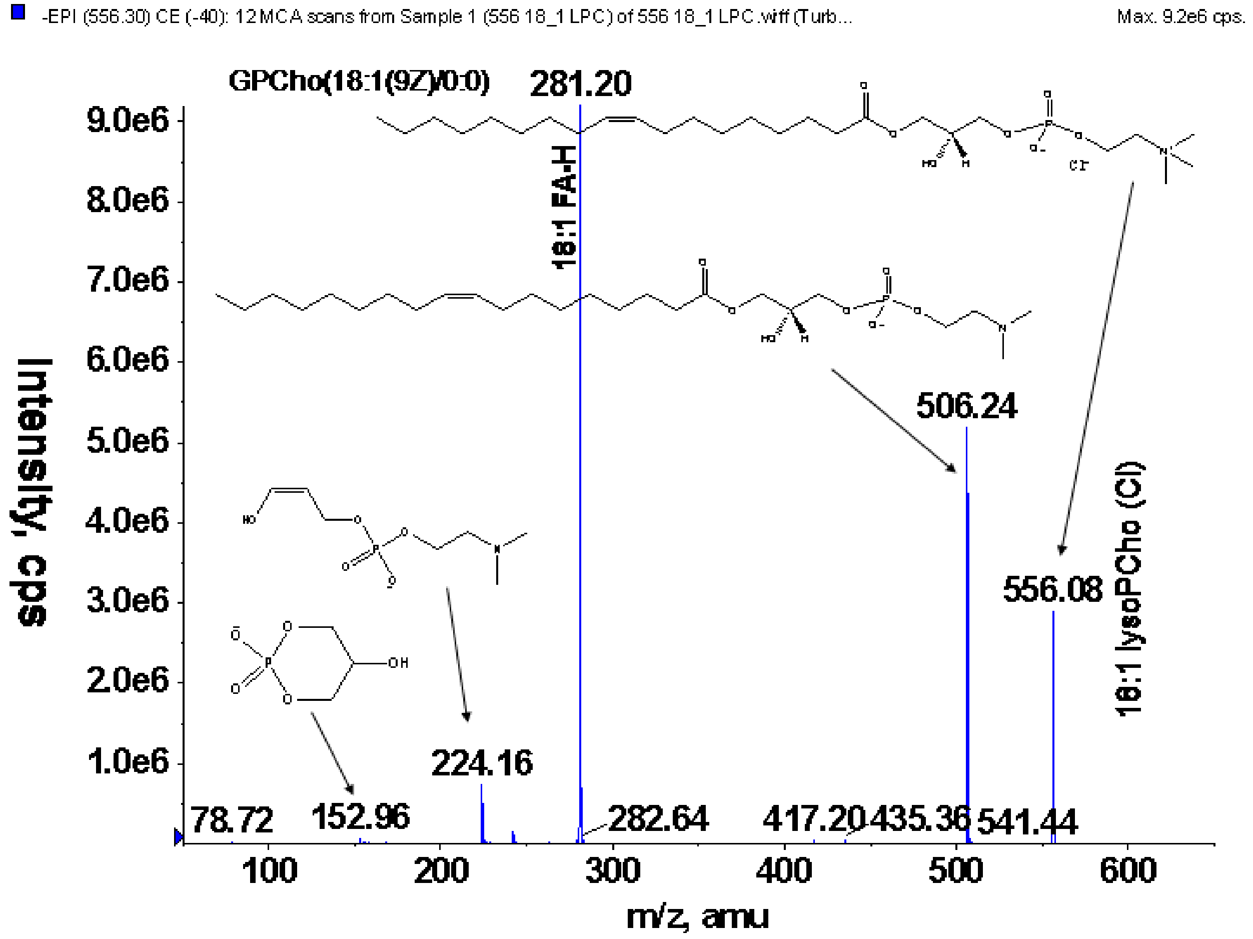

Figure S7b. Fragmentation spectrum of PC(18:1/0:0) in negative ionization.

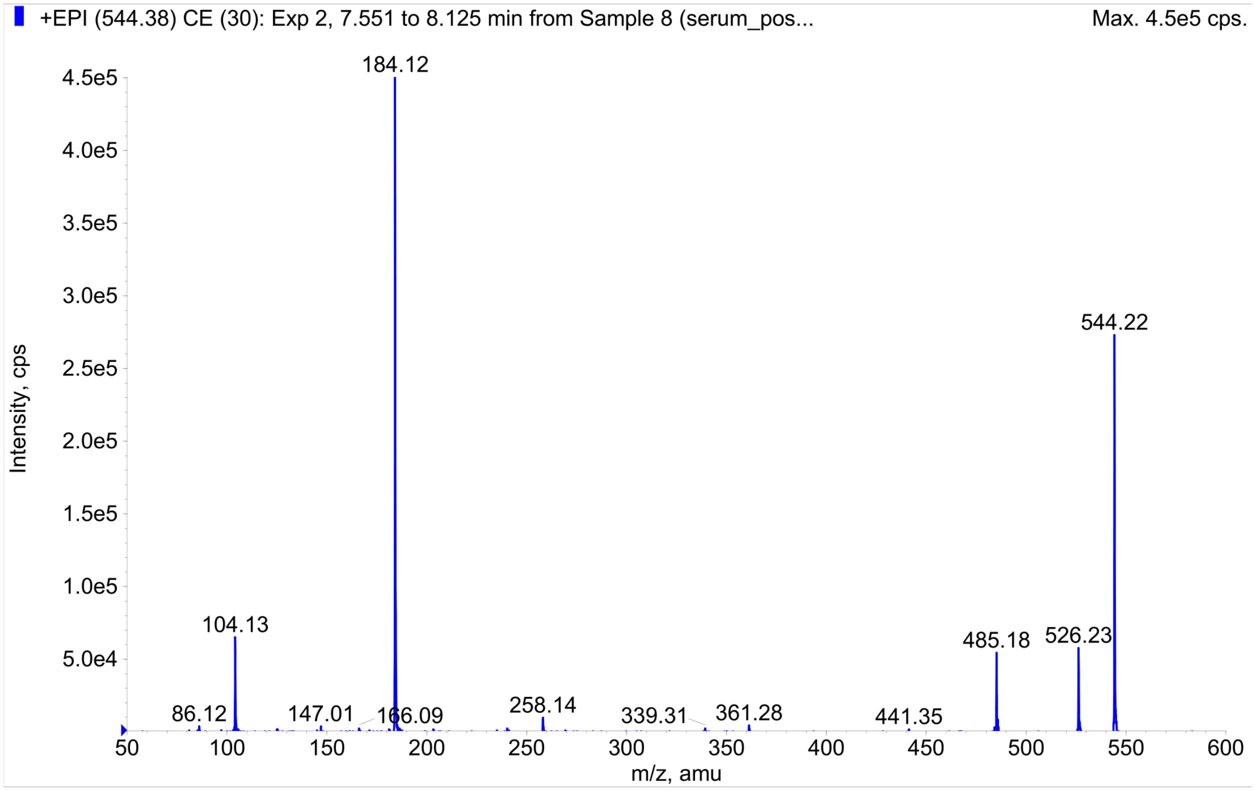

Figure S8a. Fragmentation spectrum of m/z 544.38 in positive ionization.

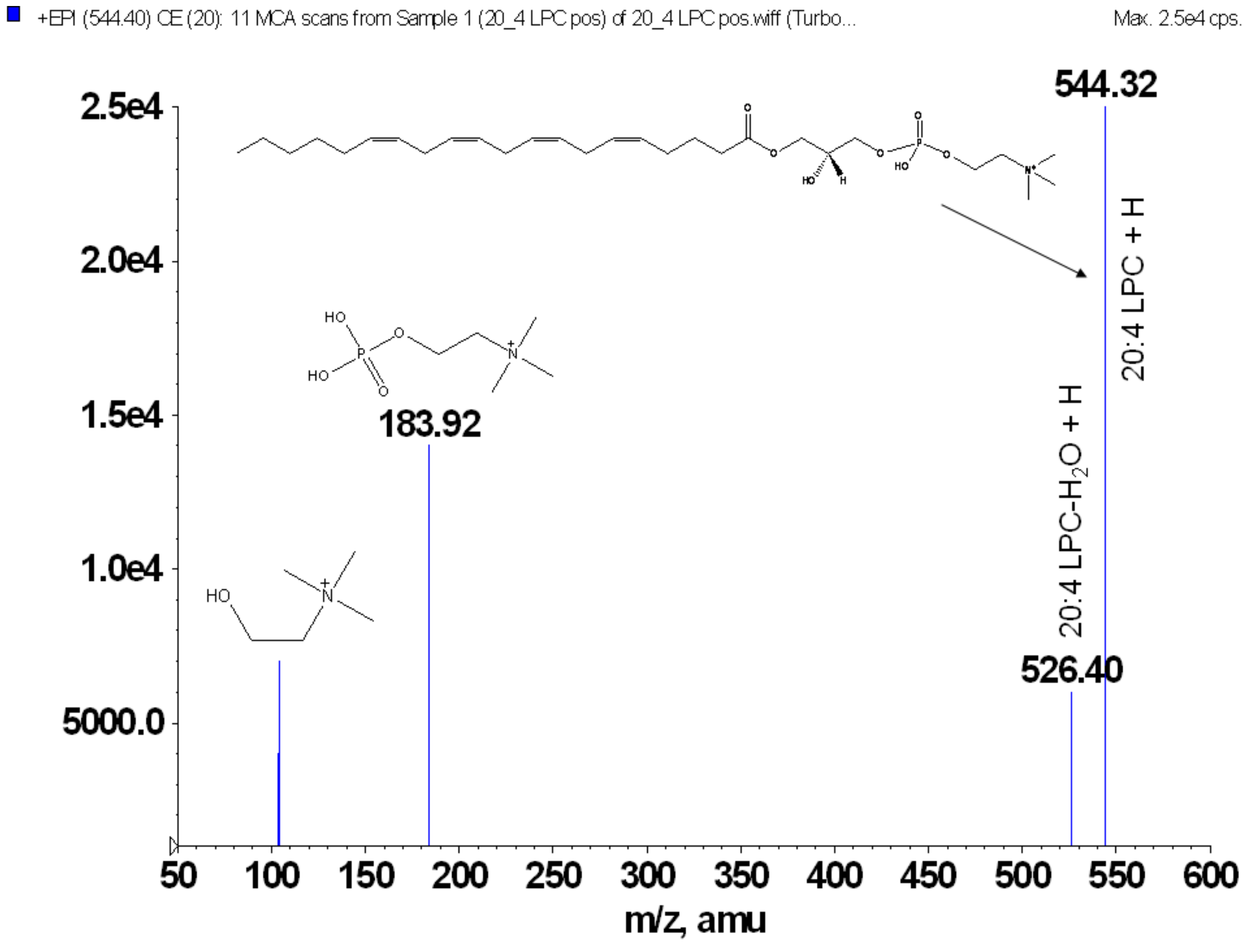

Figure S8b. Fragmentation spectrum of PC(20:4/0:0) in positive ionization.

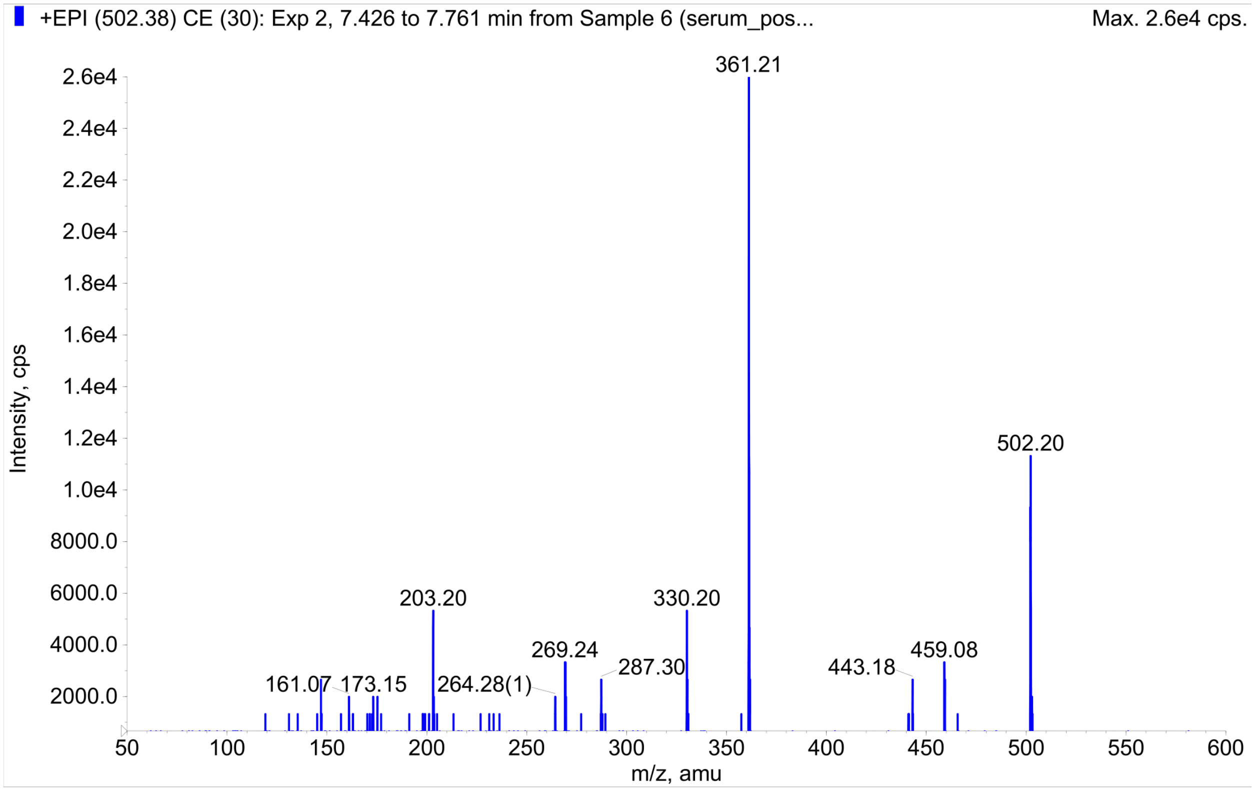

Figure S9a. Fragmentation spectrum of m/z 502.38 in positive ionization.

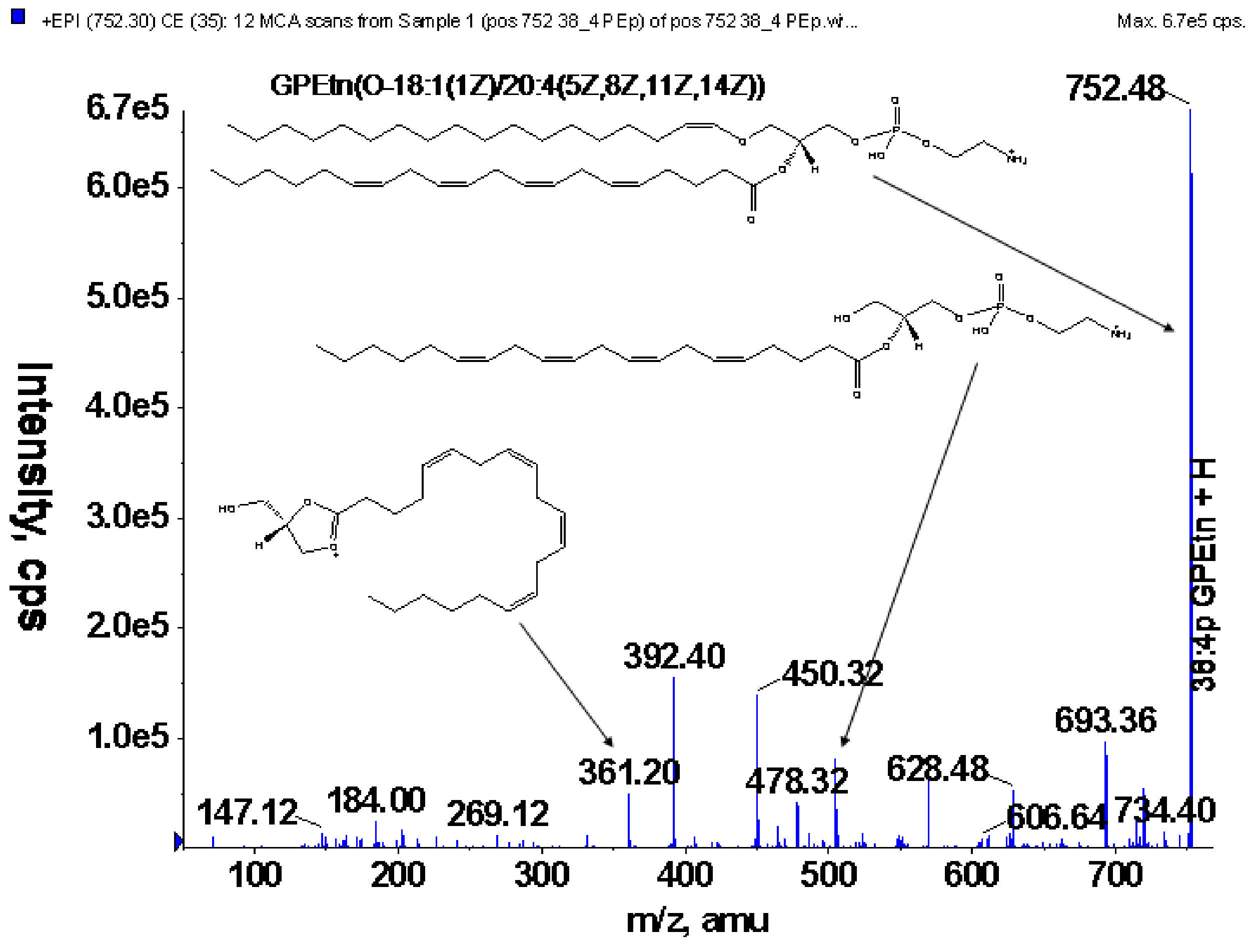

Figure S9b. Fragmentation spectrum of PE(18:1/20:4) in positive ionization.

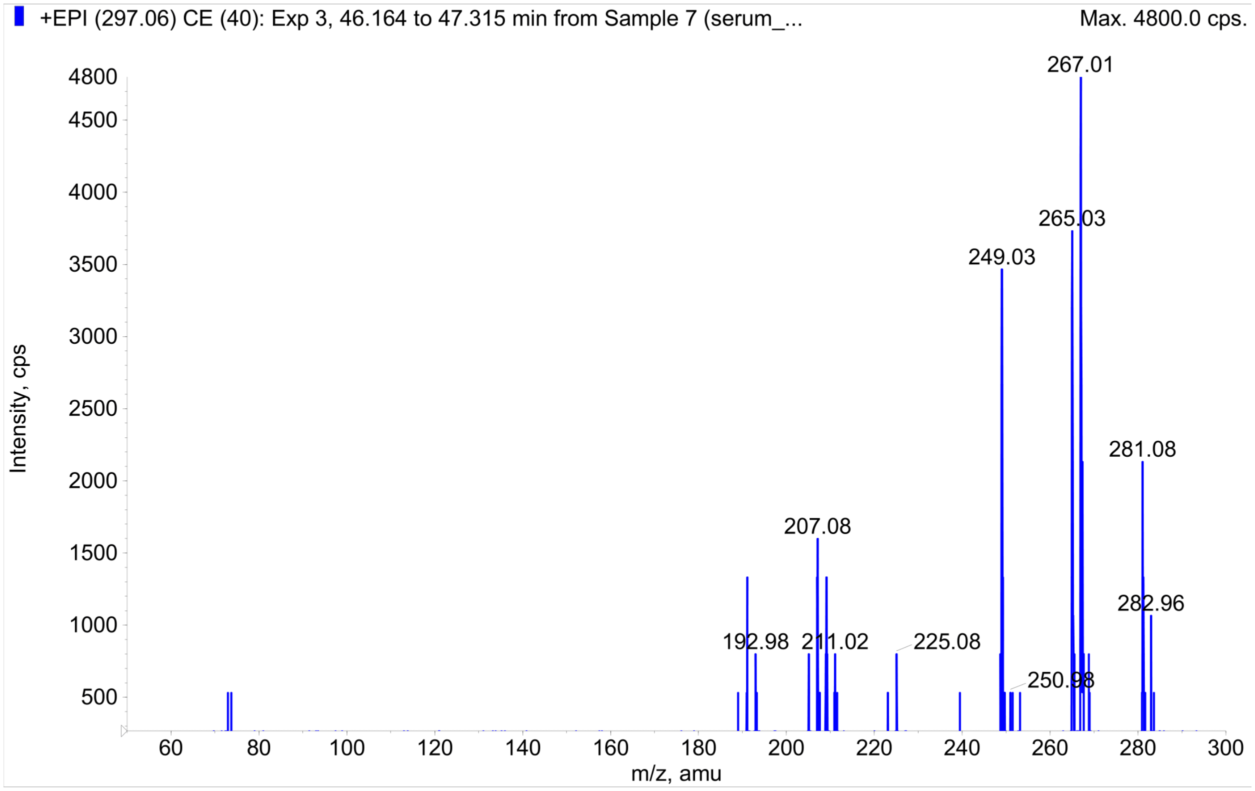

Figure S10a. Fragmentation spectrum of m/z 297.06 in positive ionization.

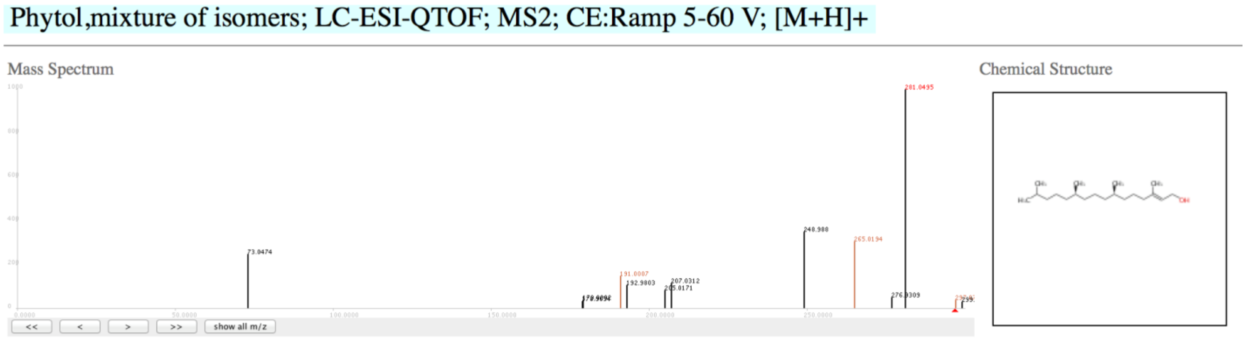

Figure S10b. Fragmentation spectrum of phytol in positive ionization.

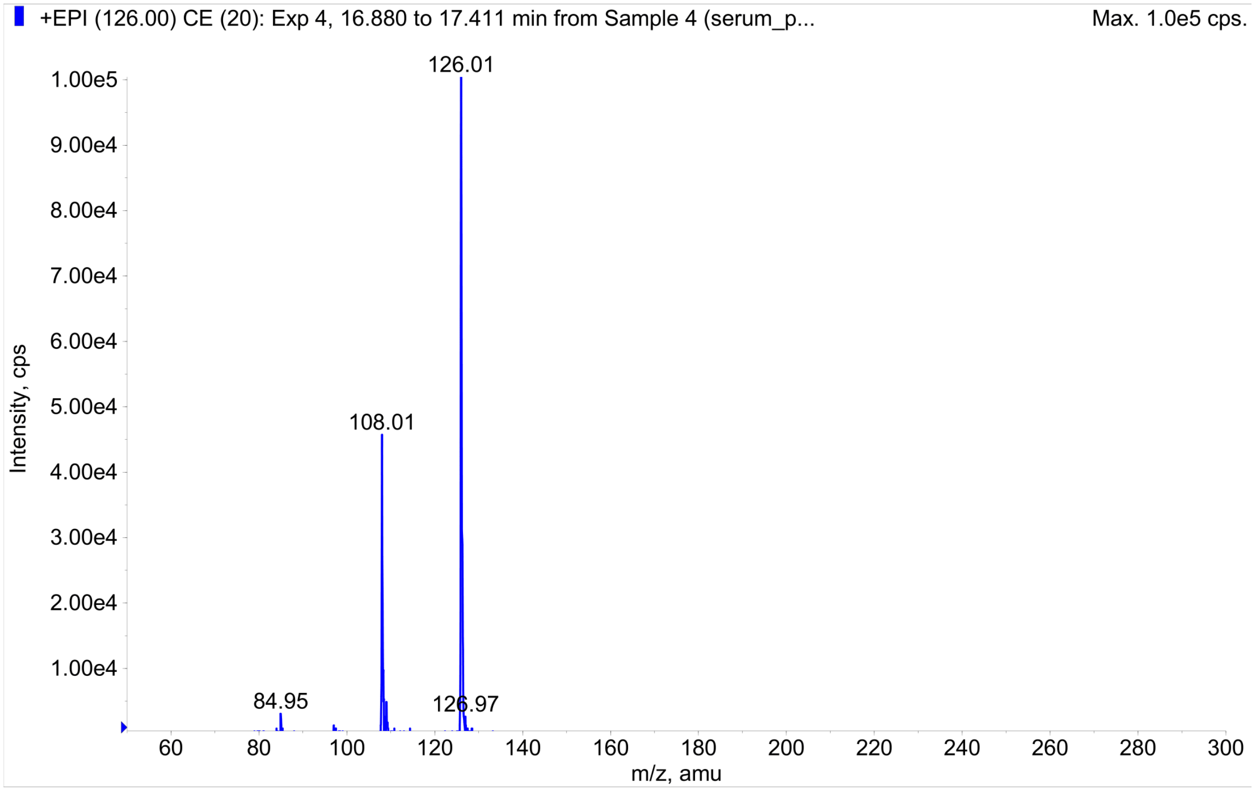

Figure S11a. Fragmentation spectrum of m/z 126 in positive ionization.

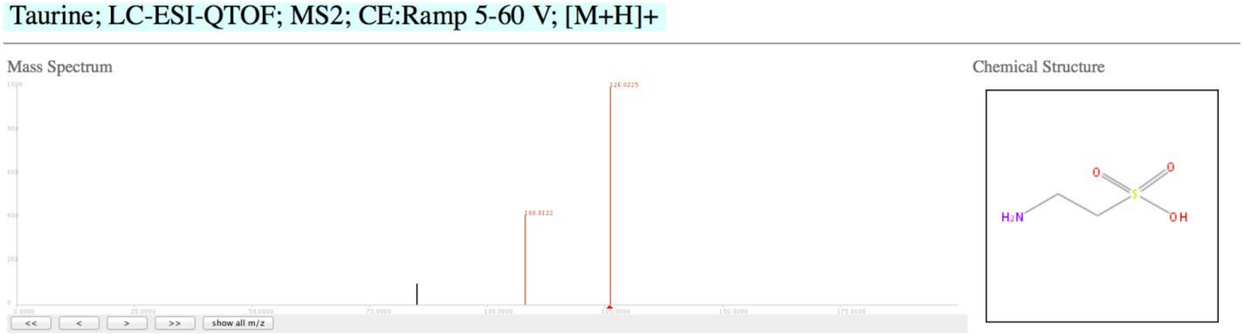

Figure S11b. Fragmentation spectrum of taurine in positive ionization.

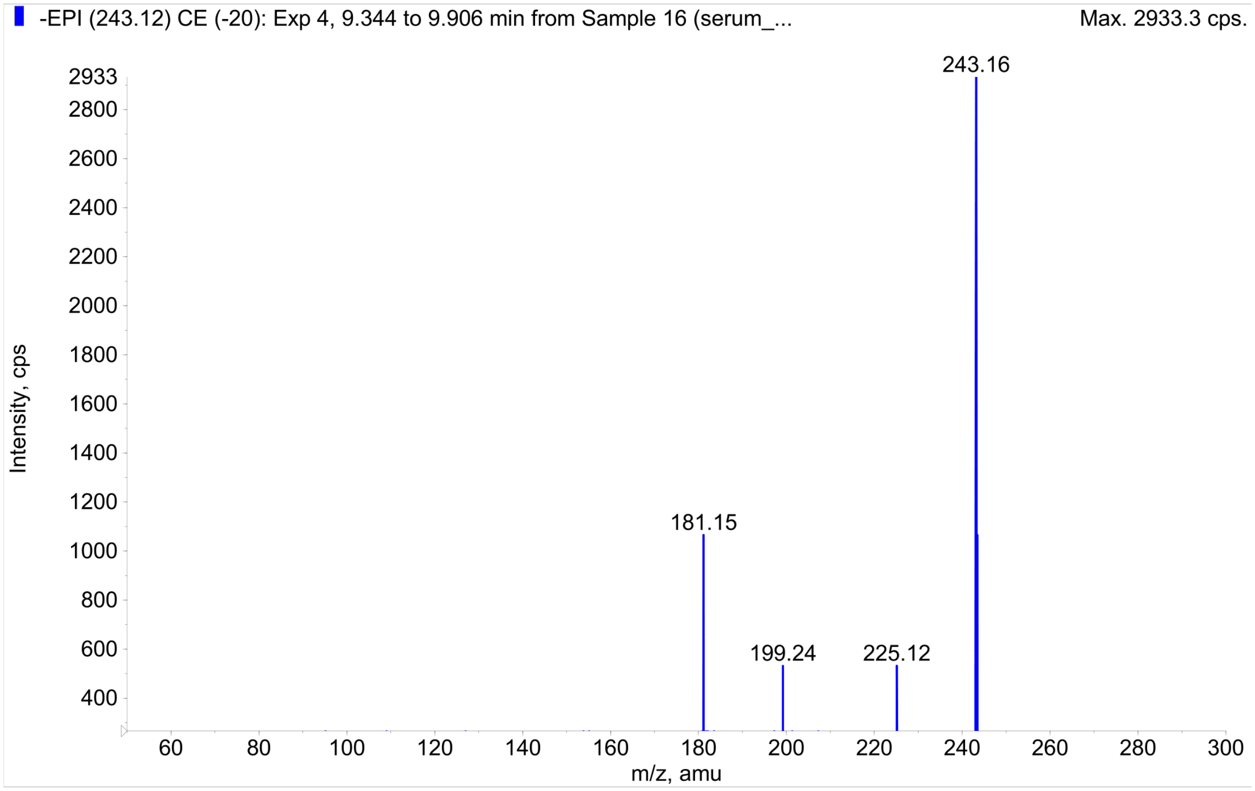

Figure S12a. Fragmentation spectrum of m/z 243.12 in negative ionization.

1,11-Undecanedicarboxylic acid (medium E)

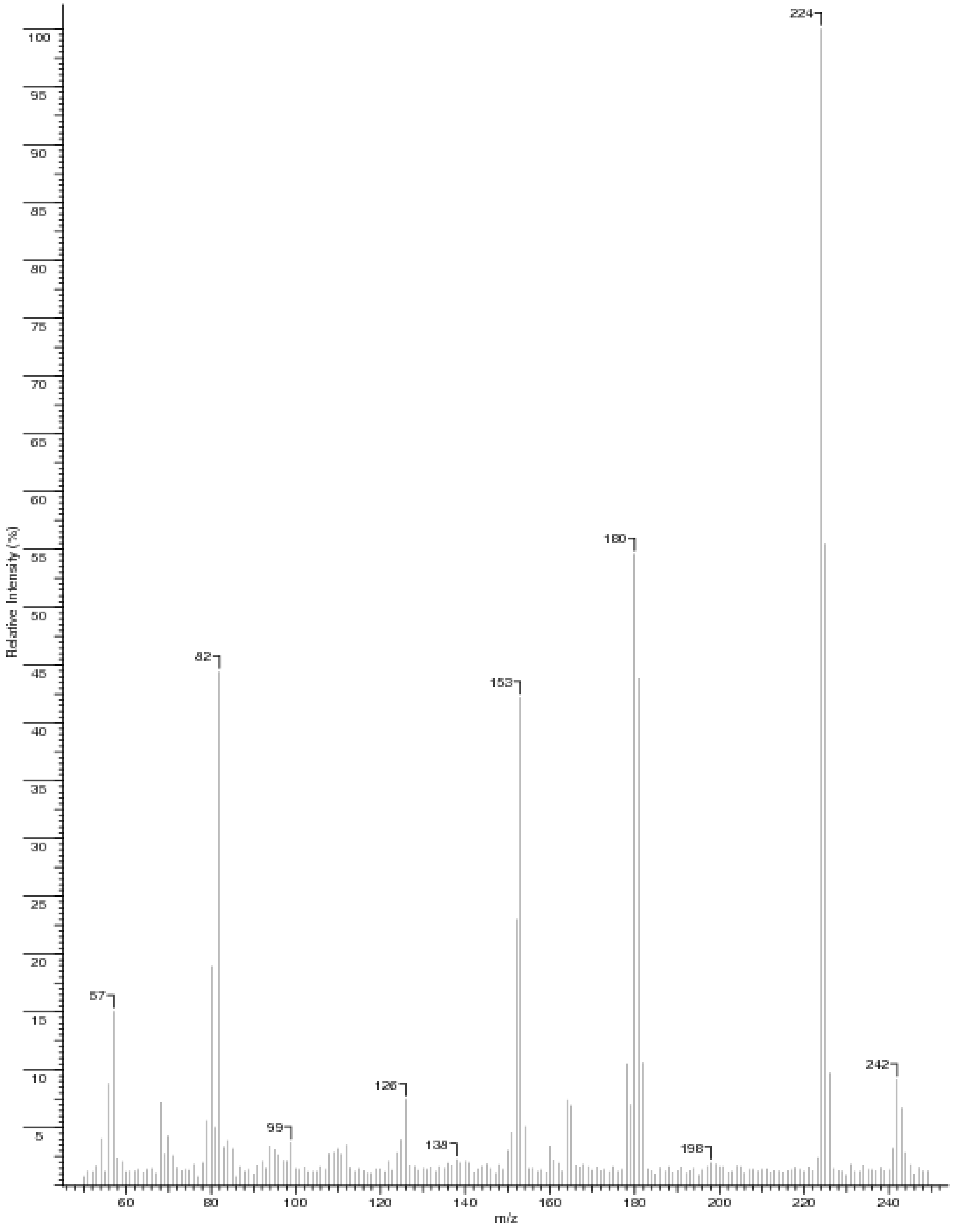

Figure S12b. Fragmentation spectrum of 1,11-undecanedicarboxylic acid in negative ionization

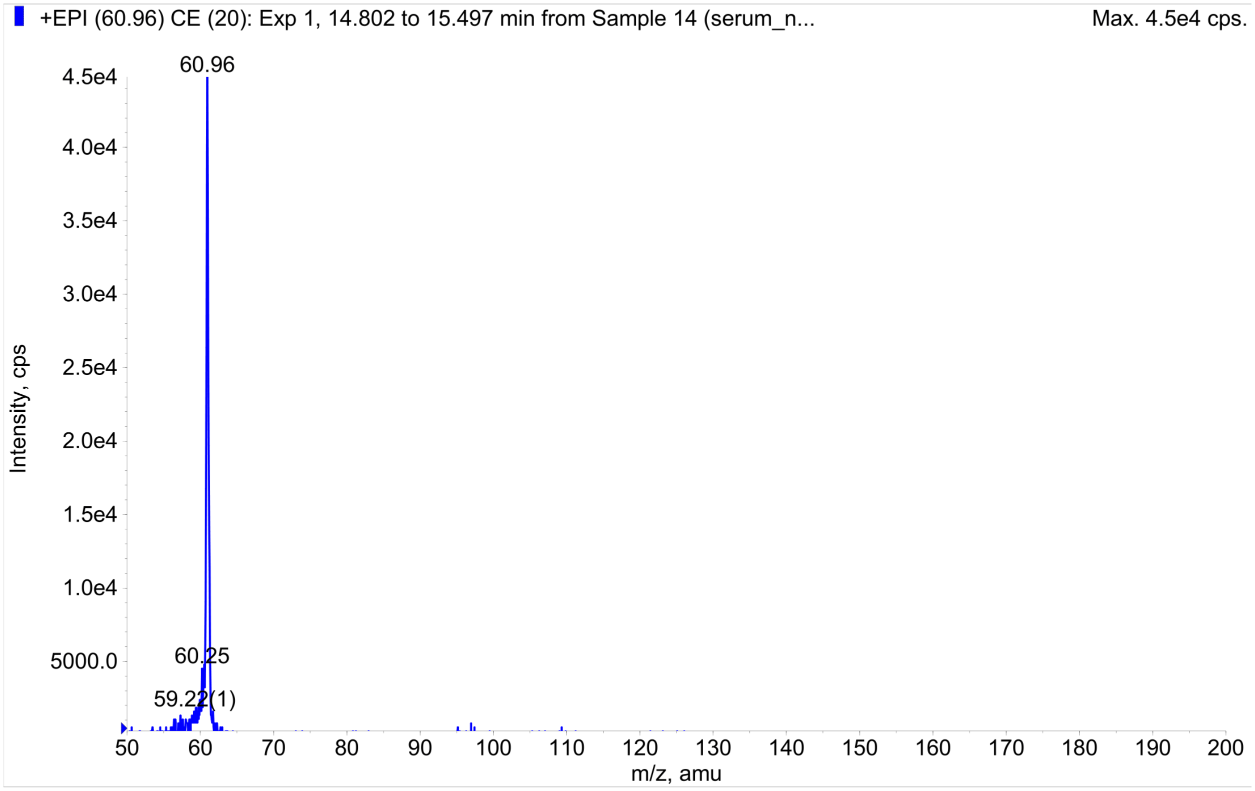

Figure S13a. Fragmentation spectrum of m/z 60.96 in positive ionization

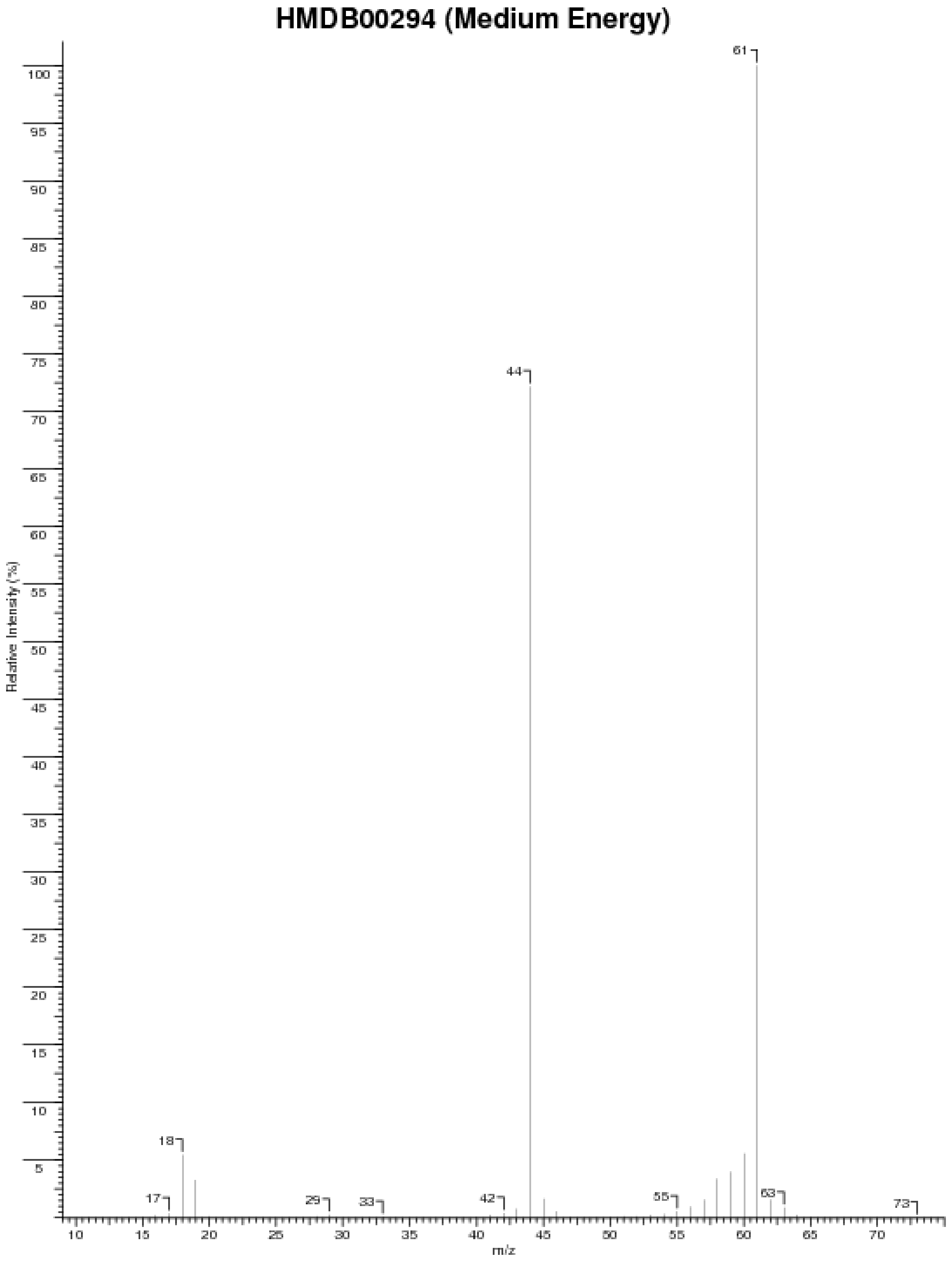

Figure S13b. Fragmentation spectrum of urea in positive ionization.
